# Dynamic Low-Rank Modulation and Frequency-Domain Collaboration for Scene-Adaptive Image Fusion Network

**DOI:** 10.3390/s26165106

**Published:** 2026-08-12

**Authors:** Yao Zhang, Lin Tian, Sirui Huang

**Affiliations:** 1 School of Electronic Engineering, Yili Normal University, Yining 835000, China; 20250050224@ylnu.edu.cn (Y.Z.); 20250050230@ylnu.edu.cn (S.H.); 2 Laboratory of Signal Intelligent Sensing and Next-Generation Communication Technology, Yili Normal University, Yining 835000, China

**Keywords:** infrared and visible image fusion, scene-adaptive, dynamic low-rank modulation, frequency-domain collaboration

## Abstract

Infrared and visible image fusion aims to integrate the complementary information from heterogeneous sensors, thereby enhancing the robustness of visual perception in complex environments. Most existing methods, however, employ fixed network parameters, a characteristic that limits their adaptive modeling capabilities for cross-modal information under scenarios such as drastic illumination changes, low light conditions, dense fog, and strong glare. To address this issue, we propose a scene-adaptive image fusion network, termed HL-Fuse, based on dynamic low-rank modulation and frequency-domain collaboration. For the spatial domain, the Hyper-LoRA is introduced via our designed SceneHyperNet, which mathematically constrains parameter variations within a low-rank subspace to adaptively calibrate attention mappings according to the global scene information. For the frequency domain, a tailored FAM is introduced to bridge spatial-domain feature aggregation and explicit spectrum reweighting by implementing targeted high- and low-frequency filtering, thereby enhancing edge and texture representation. Experiments conducted on the MSRS, TNO, M^3^FD, and FMB datasets demonstrate that HL-Fuse achieves competitive performance in terms of both multiple objective metrics and subjective visual quality, while the overall performance in the MSRS downstream object detection task is also enhanced. These results indicate the potential value of HL-Fuse for complex scene perception and remote sensing applications.

## 1. Introduction

Infrared and visible image fusion aims to integrate complementary information from heterogeneous sensors into a unified representation, thereby mitigating the perceptual limitations of single-modality systems [1,2]. In multi-source remote sensing and Unmanned aerial vehicle (UAV) systems, visible sensors can capture rich surface textures and fine-grained spatial structures, but they are highly vulnerable to low-light conditions and adverse weather. In contrast, infrared sensors can highlight thermally salient targets in complex backgrounds by capturing thermal radiation. Therefore, high-quality fused representations that combine the advantages of multiple modalities have become a key enabling technique for all-weather perception, disaster monitoring, and high-precision Earth observation.

Although deep learning has greatly advanced image fusion, especially cross-domain representation learning methods that show considerable potential in feature decoupling [3], existing approaches still face two major challenges in complex and dynamic real-world environments. First, static network parameters struggle to handle the spatiotemporal heterogeneity of all-weather, multi-source observation scenarios. Real-world illumination conditions exhibit strong non-stationary fluctuations due to factors such as day-night alternation, cloud cover, and shadows [4,5]. Most advanced fusion models employ fixed network weights and lack dynamic adaptation to heterogeneous environments, making it difficult to stably extract effective salient features under extreme illumination conditions [6,7]. Second, full-parameter updates can lead to representation drift when processing multi-source visual data with skewed distributions [8,9]. Real-world multi-scene datasets often exhibit highly imbalanced distributions in terms of illumination and meteorological conditions. When conventional fusion networks attempt to adapt to specific extreme degradation scenarios, full-parameter updates may trap the model in a “representation compromise” dilemma. Moreover, in complex non-stationary environments, such global parameter shifts are prone to introducing environment-related degradation noise, causing the network to converge to suboptimal solutions [10,11].

Recent studies have shown that dynamic network architectures and parameter modulation mechanisms provide promising solutions for improving environmental adaptability [12,13,14]. By perceiving external priors and adapting the feature space in a data-dependent manner, these models can generalize more effectively to complex scenarios. However, most existing dynamic fusion paradigms still rely on heavy global parameter generation, which may introduce optimization instability. Moreover, they are often dominated by local interactions in the spatial domain, making it difficult to simultaneously preserve effective feature disentanglement and high-frequency detail fidelity. These observations motivate us to explore a scene-aware and adaptively modulated representation paradigm for complex all-weather environments [15,16].

To this end, this paper proposes a scene-adaptive image fusion network, termed HL-Fuse, based on dynamic low-rank modulation and frequency-domain collaboration. The distinct originality of this work lies in the tailored architectural adaptation that integrates a hypernetwork-driven parameterized space with an explicit frequency-domain decoupling scheme. Unlike generic dynamic networks, HL-Fuse introduces the hypernetwork-driven dynamic low-rank modulation mechanism (Hyper-LoRA) via our designed SceneHyperNet, which mathematically constrains the dynamically generated weights within a specific low-rank subspace, thereby regulating parameter variations across continuous non-stationary illumination scenarios. Specifically, to address the spatiotemporal heterogeneity of variable observation scenarios, SceneHyperNet extracts global multimodal scene priors in real time and dynamically generates environment-specific low-rank modulation weights. This low-rank design is not merely used for parameter compression; instead, it modulates attention mappings within a constrained parameter subspace, enabling the model to obtain more stable feature representations under complex illumination and background variations. Furthermore, to alleviate texture loss and artifacts caused by purely spatial-domain fusion, the secondary originality stems from introducing a tailored Frequency-Aware Modulator (FAM), which bridges spatial-domain feature aggregation and frequency-domain spectrum reweighting by implementing targeted high- and low-frequency filtering. FAM employs the fast Fourier transform to decompose fused features into low- and high-frequency components and reweights them in the frequency domain, thereby enhancing thermally salient object boundaries and high-frequency texture details. Extensive experiments on mainstream multi-scene benchmark datasets, covering complex street scenes, all-weather autonomous driving, and nighttime surveillance, demonstrate that HL-Fuse achieves competitive overall performance in terms of visual fidelity and downstream object detection. Furthermore, the proposed framework shows adaptability under complex illumination and degraded scenes, indicating its potential for remote sensing applications such as UAV-based ground observation. In addition, the feature modulation and frequency collaboration strategies are methodologically relevant to optical measurement tasks in extreme environments, e.g., high-temperature Digital Image Correlation (DIC) [17,18]. In ultra-high-temperature mechanical testing (up to 3000 °C), deformation tracking suffers from intense background thermal radiation and illumination fluctuations. The adaptive low-rank modulation and frequency filtering schemes in HL-Fuse may offer useful insights into mitigating thermal radiation interference while preserving high-frequency speckle details in such high-temperature DIC systems.

The main contributions of this work are summarized as follows:

1. We propose a scene-adaptive image fusion network, termed HL-Fuse, based on dynamic low-rank modulation and frequency-domain collaboration. Within a dual-branch framework, we introduce a hypernetwork-driven dynamic low-rank modulation mechanism via our designed SceneHyperNet, mathematically constraining parameter variations within a low-rank subspace to resolve the optimization instability and representation drift commonly found in unconstrained dynamic networks.
2. We introduce a frequency-domain modulation module (FAM) before decoding to perform explicit spectrum reweighting. By decoupling low- and high-frequency components, FAM circumvents the local field-of-view limits of standard spatial operators, thereby enhancing structural edges and texture fidelity.
3. We conduct comprehensive evaluations on four datasets, analyze the effectiveness of each module through ablation experiments, and further validate downstream object detection performance. Experimental results demonstrate that HL-Fuse achieves competitive overall performance and shows promising application potential for image fusion tasks in complex all-weather remote sensing scenarios.

## 2. Related Work

### 2.1. Traditional Image Fusion Methods

Early infrared and visible image fusion methods mainly relied on hand-crafted feature extraction and predefined mathematical fusion rules. Multi-scale decomposition is one of the representative strategies, which transforms images into a multi-scale or multi-resolution domain using tools such as the Laplacian pyramid and the nonsubsampled contourlet transform, and then fuses the corresponding coefficients according to local energy-based rules [19,20]. In addition, sparse representation and low-rank representation improve the fidelity of fusion results by exploiting intrinsic structural priors of images [21,22,23]. Although these methods provide a certain degree of interpretability under ideal illumination conditions, they heavily depend on hand-crafted priors. When applied to complex all-weather and remote sensing observation scenarios with highly non-stationary characteristics, their fixed operators often struggle to capture nonlinear cross-modal interactions, resulting in limited robust generalization under challenging land-cover backgrounds.

### 2.2. Static Deep Image Fusion Models

With the development of deep learning, image fusion has shifted from hand-crafted feature design to data-driven representation learning. Autoencoders (AEs) and end-to-end Convolutional neural networks (CNNs) have greatly simplified the fusion pipeline by constructing deep nonlinear mappings [24,25]. In recent years, Transformer-based models and cross-domain representation learning methods have attracted increasing attention due to their strong capability for modeling global interactions. Through self-attention mechanisms, these models can capture long-range cross-modal dependencies. However, whether based on CNNs with local receptive fields or Transformers with global modeling capability, most existing fusion networks are still constrained by a train-once, static-inference paradigm. That is, once the network weights are fixed, the model cannot adapt to changes in external observation conditions during inference [26,27,28,29,30]. When the test scene differs significantly from the training distribution, fixed mappings may struggle to adapt to variations in illumination, weather, and background texture, leading to weakened local details, reduced contrast, or insufficient utilization of cross-modal information. Therefore, how to introduce an input-dependent adaptive modulation mechanism during inference remains an important research problem for image fusion in complex environments.

### 2.3. Frequency-Domain Representation and Feature Modulation

In complex all-weather multi-source observations, a core challenge of image fusion is to faithfully preserve thermally salient targets and high-frequency details, such as surface textures and small vehicles. Many deep fusion methods mainly rely on spatial attention mechanisms to allocate feature weights. Although these methods can be effective in local regions, they are constrained by finite receptive fields and often struggle to model frequency distributions and structural variations in complex degraded scenes from a global perspective. Frequency-domain analysis provides a unique physical and signal-processing perspective for cross-modal decoupling. Through the Fourier transform, an image can be decomposed into a phase component that mainly preserves spatial structural information and an amplitude component that reflects the frequency magnitude or energy distribution. Recent studies have shown that interactions in the spectral domain can enable low-cost and efficient global feature mixing [31,32,33]. Furthermore, to fully leverage the potential of frequency-domain representations for detail preservation, we introduce a FAM during the feature aggregation stage. By explicitly decoupling and reweighting the low- and high-frequency components, this modulation mechanism effectively amplifies high-frequency textures and sharpens edge structures.

### 2.4. Dynamic Networks and Task-Driven Fusion

As image fusion gradually evolves toward task-driven high-level semantic perception, establishing a dynamic bridge between low-level pixel reconstruction and high-level semantic features has become an important research direction. To overcome the limitations of traditional static representations, recent studies have explored introducing the concept of parameter-efficient fine-tuning from large-scale models into cross-modal tasks. Specifically, hypernetworks have been shown to generate conditional weights or weight offsets for the main network according to external input conditions [12,13]. Meanwhile, low-rank adaptation parameterizes model weight updates within a low-rank subspace, providing a new avenue for parameter-efficient cross-domain feature adaptation and distribution alignment [15,16]. However, existing dynamic models still face a dilemma in multimodal fusion: dynamically generating full parameters may introduce optimization instability or even training collapse [12,13,14], while simple conditional injection may be insufficient to align semantic features required by downstream tasks in complex contexts [34,35,36]. To address this dilemma, this paper explores the introduction of a Hyper-LoRA mechanism into multimodal image fusion. By mapping global multimodal scene priors to a low-rank weight subspace, this paradigm enables stable dynamic parameter calibration and enhances the model’s adaptability to different input scenes. Such dynamic calibration further improves the suitability of fused images for downstream visual tasks.

## 3. Methods

### 3.1. The HL-Fuse Framework

**Figure 1:**
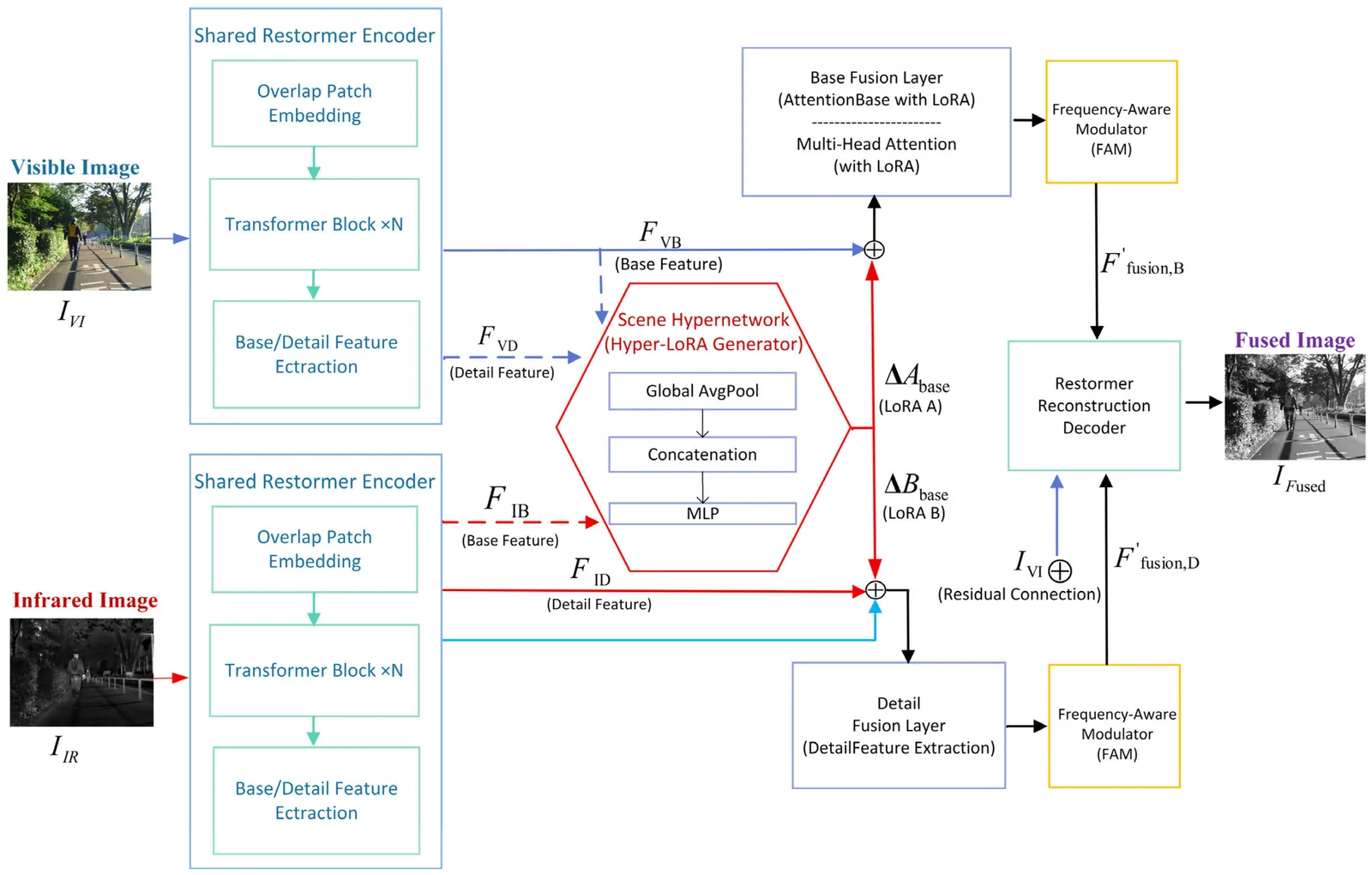
Overall framework of HL-Fuse.

To overcome the representation bottleneck of traditional static models under complex illumination conditions, we propose a scene-adaptive image fusion network, termed HL-Fuse, based on dynamic low-rank modulation and frequency-domain collaboration. As shown in Figure 1.

HL-Fuse adopts an end-to-end architecture consisting of “feature decoupling, dynamic fusion, frequency-domain enhancement, image reconstruction”. Given a strictly registered infrared image ($I_{ir}$) and visible image ($I_{vi}$), the forward process of the network can be decomposed into the following four core stages:

Dual-Branch Decoupled Feature Extraction: In the feature extraction stage, inspired by the dual-branch decoupling paradigm [30], the network constructs a pair of weight-sharing Siamese Restormer encoders to perform parallel feature extraction on the input images. To better handle multiscale spectral distributions in complex scenes, the encoder explicitly decouples the extracted latent-space features into two components: base features, designed to capture large-scale structural information and global intensity distributions, and detail features, focused on local high-frequency textures.

Scene-Adaptive Dynamic Fusion: This stage aims to achieve dynamic feature calibration based on external environmental conditions. Given the distinct frequency-band characteristics of the base and detail features, the model designs asymmetric and differentiated fusion paths. In the base feature branch, we implement the Hyper-LoRA mechanism through SceneHyperNet for the current lighting environment. These matrices are then injected into the Transformer’s self-attention maps to perform adaptive calibration by modeling the relevance of the base features to the current scene. In the detail feature branch, to avoid contamination from excessive smoothing of global weights, the model employs a nonlinear coupling module based on an inverted residual block to enhance the nonlinear representation capability of local texture information.

Frequency-Domain-Aware Enhancement Module: Before the fused features are fed into the decoder, to further mitigate the weakening of edge textures in complex backgrounds, the fused base and detail features are respectively fed into a FAM. This module maps spatial features to the frequency domain via a Fast Fourier Transform (FFT) and uses learnable frequency-band compensation parameters to explicitly decouple and reweight high- and low-frequency components. This mechanism effectively compensates for the limitations of pure spatial-domain fusion, enhancing the model’s ability to represent high-frequency edges and local texture information.

Joint Reconstruction and Global Residual Compensation: The base and detail features, after frequency-domain enhancement, undergo concatenation and dimensionality reduction along the channel dimension, and are then fed into a decoder composed of multiple Transformer modules to gradually reconstruct a pixel-level fused image. Specifically, to maximize the preservation of the source image’s natural background topology and true luminance mapping, a global residual connection ($+I_{vi}$) from the visible input image is introduced at the end of the decoder, ultimately producing a high-quality fused image that combines thermal saliency with high-fidelity texture.

### 3.2. Scene-Aware Hyper-Network and Dynamic Low-Rank Modulation

**Figure 2:**
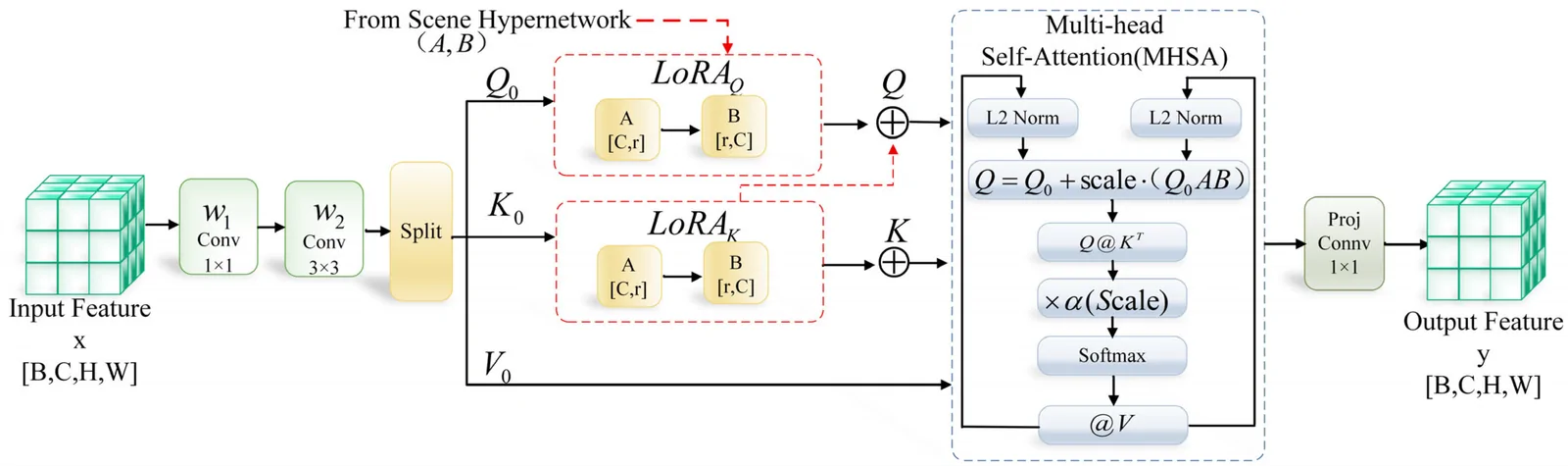
Hyper-LoRA module.

To address the representation rigidity of traditional static fusion networks when dealing with heterogeneous lighting conditions, this paper introduces a scene-aware hypernetwork and a dynamic low-rank modulation mechanism. This mechanism extracts global multimodal priors from the base features of the source images and generates modulation weights specific to the current scene in real time. As shown in Figure 2.

Let $F_{ir}$ and $F_{vi}$ denote the infrared and visible base features extracted by the encoder, respectively. To capture global environmental statistical information, SceneHyperNet first applies adaptive Global average pooling (GAP) to compress the spatial dimensions and obtain modality-specific environmental prior vectors. Subsequently, these two prior vectors are concatenated along the channel dimension to form a joint scene descriptor, which is then fed into a Multilayer perceptron (MLP) containing a Dropout layer to mitigate noise interference and dynamically generate low-rank modulation parameters. To constrain the parameter space of the dynamic modulation branch and prevent overfitting to environmental noise, the generated weight vector is reshaped into a pair of shared low-rank modulation matrices: Dimension-Reduction Matrix $A\in R^{C\times r}$ and Dimension-Increase Matrix $B\in R^{r\times C}$. Here, r represents the rank of the low-rank modulation. In this paper, we set r=16 to strike a balance between dynamic modulation capability and additional parameter overhead. During the base feature fusion and attention computation stages, let the input feature be x. Unlike traditional self-attention, which performs direct projection, feature x first undergoes successive 1×1 convolution ($W_{1}$) and 3×3 convolution ($W_{2}$) for local feature alignment, and is then split along the channel dimension to generate the initial query matrix ($Q_{0}$), key matrix ($K_{0}$), and value matrix ($V_{0}$). To suppress environmental noise in feature mapping, the generated low-rank modulation matrices A and B are injected into the mapping processes of $Q_{0}$ and $K_{0}$ to achieve scene-related dynamic modulation. To ensure mapping symmetry and distribution consistency in the feature space during attention correlation measurement, and in contrast to the static joint attention guidance mechanism in traditional methods [37], the Q and K branches share the same set of dynamic modulation weights. It should be noted that “shared” here means that the Q and K branches use the same set of low-rank modulation matrices A and B generated by SceneHyperNet. Although their input features differ, the low-rank modulation parameters remain consistent to reduce additional parameters and constrain distribution shifts in attention correlation measurement. Taking the Query matrix as an example, its dynamic update formula is

(1) $$Q=Q_{0}+\mathrm{scale}\cdot \left(Q_{0}AB\right)$$

Here, the scaling factor is formulated as $\mathrm{scale}=\frac{\alpha }{r}\times \beta$ (with α=32, r=16 and β=2.5). The $\frac{\alpha }{r}$ term normalizes activation variance to ensure gradient stability, whereas the amplification coefficient β provides sufficient leverage for the dynamic branch to handle extreme degradation without modifying the frozen weights $Q_{0}$. The Key matrix K follows identical logic. This properly calibrated low-rank constraint prevents optimization instability commonly caused by unconstrained full-dimensional dynamic weights.

In the core computation of Multi-Head Self-Attention, to further stabilize feature distributions under extreme conditions, the model applies L2 normalization to the modulated Q and K matrices, then computes the attention matrix and introduces an independent attention scaling factor (α) for adjustment. The features aggregated through self-attention are finally passed through the projection layer for output. This dynamic calibration mechanism helps improve the stability of cross-modal feature fusion in complex scenarios, such as extremely dark shadows or local overexposure.

### 3.3. Frequency-Aware Modulator

**Figure 3:**
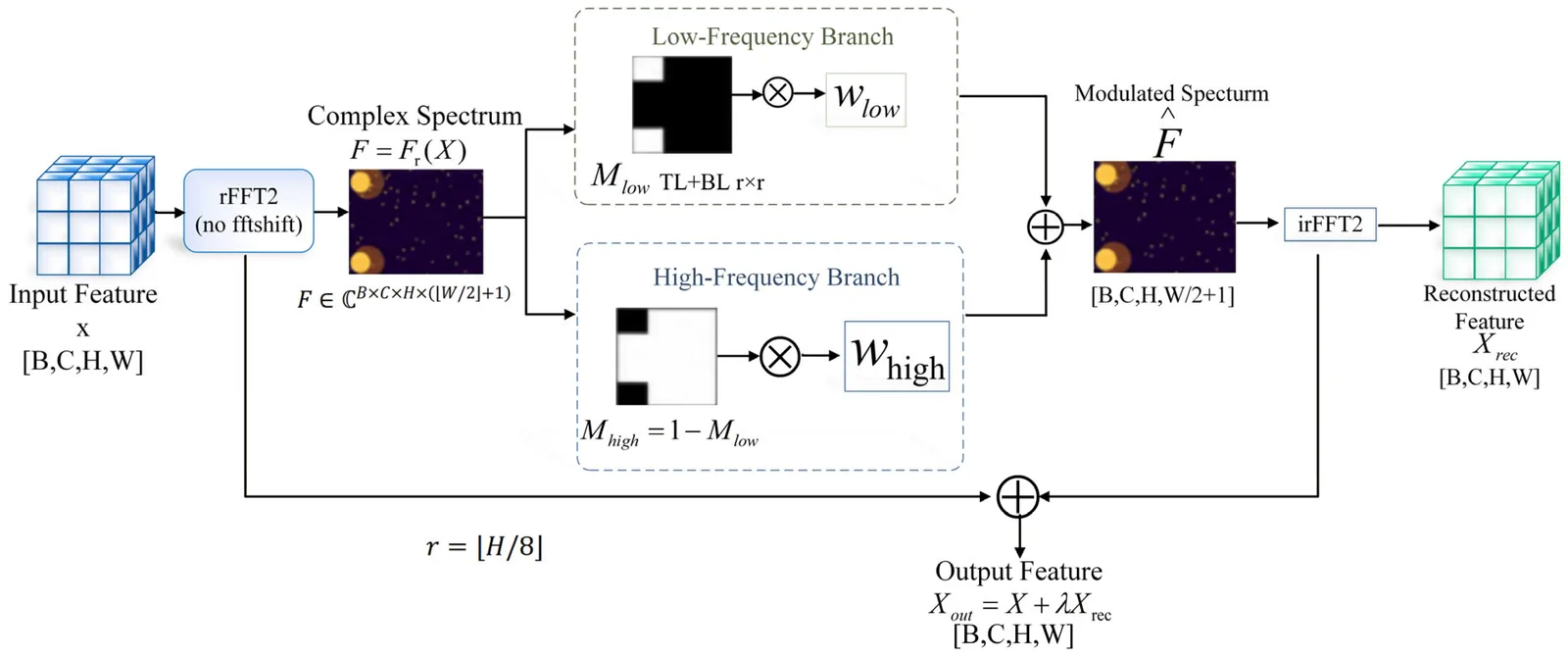
FAM module.

After dynamic feature fusion, to further eliminate interference from complex land-cover backgrounds and explicitly enhance the boundaries and high-frequency textures of salient objects, we introduce the FAM. Unlike traditional methods that extract details through local convolution in the spatial domain, FAM provides an efficient feature enhancement paradigm with a global receptive field by performing explicit spectral decoupling and reweighting in the frequency domain, as shown in Figure 3.

Given the input fused features,

(2) $$X\in R^{B\times C\times H\times W}$$

FAM first converts the features to float32 to improve the numerical stability of the Fourier transform computation. Subsequently, a two-dimensional real fast Fourier transform is used to map the spatial features to the frequency domain:

(3) $$F=F_{r}(X)$$

where $F_{r}(\cdot )$ denotes a two-dimensional rFFT operation. Since rFFT retains only the non-negative frequency components in the width direction, the dimensions of the spectrum (F) are given by

(4) $$F\in C^{B\times C\times H\times \left(\left\lfloor W/2\right\rfloor +1\right)}$$

In the implementation, no fftshift operation is performed on the spectrum. Instead, the low- and high-frequency masks are constructed directly in the rFFT half-spectrum coordinates. Let the low-frequency cutoff scale be

(5) r=⌊H/8⌋

It is crucial to note that the frequency cutoff threshold r is not a static absolute pixel value, but dynamically scales with the spatial height H of the input feature map during each forward pass, inherently ensuring generalization across varying image resolutions. Since the rFFT output contains only non-negative frequencies in the width direction, while the height direction retains a periodic distribution of both positive and negative frequencies, the low-frequency region is distributed simultaneously in the top-left and bottom-left corners of the spectrum. Therefore, this paper constructs the low-frequency mask as follows:

(6) $$M_{low}[:,:,0:r,0:r]=1$$

(7) $$M_{low}[:,:,H-r:H,0:r]=1$$

All other positions are set to 0. The high-frequency mask is defined as

(8) $$M_{high}=1-M_{low}$$

The above configuration allows the low-frequency mask to simultaneously cover both the positive and negative low-frequency regions along the vertical frequency axis, thereby adapting to the frequency distribution under the rFFT half-spectrum representation. Subsequently, FAM reweights the spectrum using two learnable channel-wise frequency-band compensation parameters, $\omega_{low}$ and $\omega_{high}$:

(9) $$\hat{F}=(\omega_{low}⊙M_{low}⊙F)+(\omega_{high}⊙M_{high}⊙F)$$

where ⊙ denotes element-wise multiplication. $\omega_{low}$ and $\omega_{high}$ are learnable channel-level compensation parameters with a size of 1×C×1×1, and they are applied to all spectral positions of the corresponding channels during computation. Furthermore, $\omega_{low}$ and $\omega_{high}$ are merely initialized to 1.1 and 1.5, respectively. Rather than being fixed constants, these higher initial values for the high-frequency component provide a mild high-frequency enhancement prior for the model, which helps preserve edge and texture responses in the early stage of training. As training proceeds, the final band weights are adaptively updated by the network according to the data distribution. The modulated spectrum is then mapped back to the spatial domain via a two-dimensional inverse real Fourier transform. As training proceeds, the final band weights are adaptively updated by the network according to the data distribution. The modulated spectrum is mapped back to the spatial domain via a two-dimensional inverse real Fourier transform:

(10) $$X_{rec}=F_{r}^{-1}(\hat{F})$$

Finally, to control the injection strength of the frequency-domain enhancement branch, this paper adopts residual aggregation to fuse the frequency-domain reconstructed features with the original input features:

(11) $$X_{out}=X+0.8X_{rec}$$

Here, η is the residual injection coefficient, which is set to 0.8 in this paper. This residual aggregation method introduces frequency-domain compensation information while preserving the original spatial features, thereby preventing the frequency-domain reconstructed features from completely overwriting the original representation. The FAM outputs are then fed into the decoder, where they undergo channel-reduction convolution, Transformer modules, and output mapping to complete fused image reconstruction, thereby enhancing the edge and texture information in the fused features.

### 3.4. Training Objectives

This paper adopts a two-stage self-supervised training strategy. The first stage is the source image self-reconstruction pretraining stage, which is used to initialize the encoder and decoder. The second stage is the fusion optimization stage, in which the fusion module participates in end-to-end training. Since the main improvements in this paper focus on dynamic low-rank modulation and the frequency-domain collaboration module, while the loss function is mainly used as a training constraint, this section primarily presents the optimization objective for the fusion stage.

Given an infrared image ($I_{ir}$), a visible luminance channel ($I_{vi}^{Y}$), and a fused image ($I_{fus}$), this paper draws on the intensity constraint, gradient constraint, and correlation-driven feature decoupling constraint from existing self-supervised fusion methods [25,30,32], and further introduces a standard-deviation statistical loss to jointly form the training objective for the fusion stage. The image-level loss for the fusion stage is expressed as

(12) $$L_{fusion}=L_{in}+\lambda_{g}L_{grad}+\lambda_{s}L_{sd}$$

In particular, the pixel intensity loss ($L_{in}$) is constructed using a maximum-intensity objective:

(13) $$L_{in}=\frac{1}{HW}\sum_{i=1}^{H}\sum_{j=1}^{W}\left|I_{fus}\left(i,j\right)-\max\left(I_{vi}^{Y}\left(i,j\right),I_{ir}\left(i,j\right)\right)\right|$$

This loss uses the larger pixel responses in the infrared image and the visible luminance image as self-supervised targets to preserve thermal target information in the infrared modality and high-brightness structural information in the visible modality.

To enhance the ability of the fused image to preserve the edge structures of the source images, this paper employs the absolute Sobel gradient response to construct the gradient constraint:

(14) $$L_{grad}=\frac{1}{HW}\sum_{i=1}^{H}\sum_{j=1}^{W}\left|G\left(I_{fus}\right)\left(i,j\right)-\max\left(G\left(I_{vi}^{Y}\right)\left(i,j\right),G\left(I_{ir}\right)\left(i,j\right)\right)\right|$$

In particular, $G\left(I\right)=\left|S_{x}\left(I\right)\right|+\left|S_{y}\left(I\right)\right|$, where $S_{x}\left(\cdot \right)$ and $S_{y}\left(\cdot \right)$ represent the horizontal and vertical Sobel convolution responses, respectively. This term uses the stronger gradient responses from the two source images as structural targets, thereby alleviating the edge attenuation issue in the fusion result.

The standard-deviation statistical loss constrains the fused image from the perspective of the global grayscale distribution and is expressed as follows:

(15) $$L_{sd}={\left(\sqrt{Var\left(I_{fus}\right)+\epsilon }-\gamma \sqrt{\max\left(Var\left(I_{vi}^{Y}\right),Var\left(I_{ir}\right)\right)+\epsilon }\right)}^{2}$$

Here, Var(⋅) represents the image variance, $\epsilon =10^{-6}$ is used for numerical stability, and γ=1.1. This loss function constrains the standard deviation of the fused image so that its grayscale dynamic range remains consistent with the stronger global contrast in the source images.

Furthermore, to maintain the decoupling relationship between base and detail features, this paper employs a correlation-driven feature decoupling loss ($L_{decomp}$). The optimization objective for the final fusion stage is

(16) $$L_{final}=\lambda_{f}L_{fusion}+\lambda_{d}L_{decomp}$$

In the experiments, the following parameters were set: $\lambda_{g}=60$, $\lambda_{s}=35$, $\lambda_{f}=3.0$ and $\lambda_{d}=1.0$. Due to differences in the original numerical scales of the various loss terms, this paper uses fixed weights to balance the magnitudes of the loss terms, enabling pixel response, edge structure, and global grayscale distribution to collectively participate in the optimization process. All weights are kept fixed across different experiments, and no loss function is used during testing.

## 4. Experiment

### 4.1. Datasets and Implementation Details

To comprehensively validate the scene adaptability of HL-Fuse under complex all-weather illumination conditions and its performance gains for downstream vision tasks, we conducted a systematic evaluation on four widely recognized public benchmark datasets in the field of multimodal image fusion: Multi-Spectral Road Scenarios (MSRS [38]), Netherlands Organisation for Applied Scientific Research (TNO [39]), Multi-Modal Multi-Scene Fusion Dataset (M^3^FD [40]), and Full-time Multi-modality Benchmark (FMB [41]). The network was trained on the MSRS training set (1083 pairs), while the MSRS test set (361 pairs), TNO (25 pairs), M^3^FD (300 pairs), and FMB (280 pairs) were used as test datasets to comprehensively validate the fusion performance. These four datasets have distinctive characteristics and collectively form a complete evaluation framework ranging from low-level visual fidelity to high-level semantic perception. MSRS contains drastic illumination fluctuations caused by day-night transitions, making it an ideal benchmark for validating the robustness of Hyper-LoRA dynamic weight modulation. In addition, owing to its rich traffic semantic annotations, it was selected as the core testbed for evaluating the performance gains in downstream object detection with You Only Look Once version 5 small (YOLOv5s). TNO contains complex backgrounds and sensor noise, and is mainly used to evaluate the model’s ability to extract thermally salient targets, such as camouflaged individuals, under extremely low-light conditions. M^3^FD covers a wide range of meteorological conditions, such as cloudy and foggy weather, and is used to systematically evaluate the visual fidelity of the model across diverse urban scenarios. FMB includes heterogeneous environmental degradation conditions such as rain and fog, focusing on verifying the feature stability and generalization capability of the network under harsh all-weather conditions.

The proposed HL-Fuse network is implemented using the Pytorch2.10.0+CUDA12.8 deep learning framework. Model training and ablation experiments were conducted on a single NVIDIA RTX 5090 GPU (NVIDIA Corporation, Santa Clara, CA, USA, rented from the AutoDL cloud computing platform). For cross-method comparison experiments and the downstream YOLOv5s object detection task, all comparison methods and the proposed method were uniformly deployed on a single NVIDIA RTX 5060 GPU (NVIDIA Corporation, Santa Clara, CA, USA, equipment purchased through official channels) for testing, ensuring that different methods were compared under the same inference environment. During the data preprocessing stage, the training images were pre-cropped into 128×128 image blocks with a stride of 200. Specifically, to avoid potential interference from color channels on cross-modal feature alignment, we implemented a strict color-to-grayscale conversion procedure to ensure that the network consistently focuses on learning spatial topology and texture structures within a pure luminance space, namely the Y channel.

Regarding the network optimization strategy, model training adopts a two-phase progressive optimization paradigm. The first 30 epochs constitute the feature self-reconstruction phase, during which the encoder learns the base and detail feature representations of infrared and visible images through Structural Similarity Index Measure (SSIM) loss, pixel-level loss, gradient constraints, and feature correlation constraints. This is followed by an end-to-end fusion training phase, which continues until the 100th epoch. To maximize training stability, we employed the Adam with Decoupled Weight Decay (AdamW) optimizer with weight decay ($weight\_decay=1\times 10^{-4}$) [42]. To accommodate the convergence characteristics of different modules, this paper adopts a grouped learning-rate strategy: the learning rates for the encoder and decoder are set to $5\times 10^{-6}$, the learning rates for the base and detail feature fusion layers are set to $2\times 10^{-5}$, and the learning rates for the relevant parameters of Hyper-LoRA and FAM are set to $1\times 10^{-4}$. In addition, the StepLR learning-rate decay strategy is adopted during training, reducing the learning rate to 50% of its original value every 30 epochs, with the gradient clipping threshold set to 0.5 to enhance training stability. Regarding the joint loss configuration, the fusion stage employs the image-level fusion loss and feature decoupling loss defined earlier for joint optimization, with the corresponding loss weights remaining consistent across all experiments. During the testing phase, this paper uses the model weights obtained from training to generate fused images on the MSRS test set, and further applies the same trained model weights directly to the TNO, M^3^FD, and FMB datasets to evaluate the cross-dataset generalization ability of the model. Subsequently, the generated fused images are input into a pretrained YOLOv5s object detection framework [43] to further analyze the impact of the fusion results on downstream object detection tasks.

### 4.2. Comparative Methods and Objective Evaluation Metrics

#### 4.2.1. Selection of Comparison Methods

To comprehensively evaluate the effectiveness of the proposed HL-Fuse model, this paper conducts qualitative and quantitative comparisons between HL-Fuse and eight representative infrared and visible image fusion methods under the same hardware and software environments. These methods comprehensively cover multiple advanced fusion paradigms, ranging from pure convolutional neural networks to Transformers and low-rank representations. The eight compared methods are described in detail below:

DeFusion (2022) [25]: Originating from the open-source project Decomposition ForFusion, DeFusion is a fusion method based on self-supervised image decomposition. Within a neural network framework, this method decouples source images into different intrinsic feature components, such as the base layer and detail layer, enabling efficient reorganization of cross-modal features and demonstrating high stability in background smoothing regions.

LRRNet (2023) [23]: LRRNet is a lightweight end-to-end fusion network guided by low-rank representation learning. This method decomposes source images into low-rank and sparse components in the feature space and is optimized using a multi-level loss function that progresses from details to semantics. It is representative of mathematical-prior-driven deep fusion.

SwinFusion (2022) [27]: SwinFusion is a cross-domain fusion method for modeling long-range dependencies based on the Swin Transformer architecture. It effectively utilizes a sliding-window self-attention mechanism to achieve efficient local-to-global feature aggregation while controlling computational complexity.

DATFuse (2023) [28]: DATFuse is a Transformer-based image fusion network utilizing a dual-attention mechanism. By alternately deploying channel-wise and spatial self-attention, this model deeply captures cross-modal global contextual information, significantly alleviating the limitations of traditional CNN receptive fields.

YDTR (2023) [29]: YDTR is an architecture based on a Y-shaped dynamic Transformer. This method utilizes a dual-branch structure to extract multimodal independent features and, through the dynamic Transformer module, simultaneously captures both local abrupt changes and global macro-level context at the feature level.

CDDFuse (2023) [30]: CDDFuse is a dual-branch feature decoupling and fusion network that combines the local perception of CNNs with the global interaction of Transformers. By establishing a cross-modal joint encoding paradigm, this method effectively separates images into base and detail features, and introduces a long-range self-attention mechanism during the feature fusion stage to handle global dependencies, making it a representative advanced baseline in the current direction of cross-modal feature decoupling.

DDBFusion (2024) [44]: DDBFusion is an advanced multimodal image fusion framework based on dual decomposition and Bézier curves. This method precisely separates the spatial and frequency attributes of images by introducing a dual decomposition mechanism and utilizes learnable Bézier curves to perform fine-grained feature mapping.

Conti-Fuse (2025) [10]: Conti-Fuse is a recent advanced framework based on continuous feature decomposition. This method overcomes the architectural bottlenecks of traditional discrete feature separation and effectively mitigates feature crosstalk during cross-modal information extraction by constructing a continuous feature-space mapping mechanism.

In our evaluation, all comparison methods were tested using their official open-source pretrained weights and default inference parameters. Considering that the training datasets used by the baseline models may vary, this setup directly tests the actual performance of all methods on the given test sets. To ensure a unified benchmarking environment, all test images were subjected to the same preprocessing pipeline. Specifically, regarding image resizing, all test images were fed into the networks at their original spatial resolutions without any cropping or scaling operations. Regarding the quantitative evaluation protocol, since different baseline methods adopt various color spaces (e.g., RGB) for inference, all finalized fusion results along with the source images were converted to single-channel grayscale images during the metric calculation phase. This unified evaluation color space eliminates potential assessment discrepancies caused by different color processing colorways or structural evaluation biases, ensuring a fair and objective benchmarking environment. Furthermore, the training datasets and settings for all baseline methods are summarized in Appendix A (Table A1).

#### 4.2.2. Objective Evaluation Metrics and Criteria

During the quantitative evaluation phase, to accurately quantify fusion performance across multiple dimensions, including image contrast, richness of local details, frequency-domain feature activity, absorption and transmission of complementary information, and human visual perception, we adopted a comprehensive evaluation system comprising eight objective metrics [45]. It should be noted that, to ensure the rigor and impartiality of the evaluation and to avoid structural and luminance measurement biases caused by color channels, this paper strictly enforces a unified conversion logic at the code level before calculating all objective metrics, converting all color-fused images and source images output by the models into grayscale images. Furthermore, to ensure consistency in metric computation, the evaluation process represents the images within a unified 8-bit grayscale range. The specific definitions of each metric are as follows. For all metrics, higher values indicate better information content, contrast, and detail representation in the fused image:

1. Entropy (EN) quantifies the information richness contained in a fused image based on information theory, reflecting the gradation achieved after the image’s dynamic range has been expanded. It is calculated as follows:
  (17) $$EN=-\sum_{l=0}^{L-1}p\left(l\right)\log_{2}p\left(l\right)$$
  Here, L is the total number of gray levels, usually 256, and p(l) represents the normalized probability of gray level l in the image histogram.
2. Standard deviation (SD) measures the degree of dispersion of pixel values in the fused image and directly reflects the overall contrast distribution of the image. This metric has a certain correspondence with the standard-deviation statistical constraint adopted in this paper:
  (18) $$SD=\sqrt{\frac{1}{H\times W}\sum_{i=1}^{H}\sum_{j=1}^{W}{\left(I_{fus}\left(i,j\right)-\mu \right)}^{2}}$$
  Here, H and W denote the image dimensions, and μ denotes the average grayscale value of the fused image $I_{fus}$.
3. Average gradient (AG) reflects the rate of contrast change in fine image details and is a key metric for measuring edge sharpness and clarity. This metric can be used to assess edge clarity and local detail preservation in the fused image:
  (19) $$AG=\frac{1}{HW}\sum_{i=1}^{H}\sum_{j=1}^{W}\sqrt{\frac{G_{x}{\left(i,j\right)}^{2}+G_{y}{\left(i,j\right)}^{2}}{2}}$$
  Here, $G_{x}\left(i,j\right)$ and $G_{y}\left(i,j\right)$ represent the horizontal and vertical finite-difference gradients of the image, respectively.
4. Spatial frequency (SF) evaluates the overall activity and high-frequency details of an image by combining row frequency (RF) and column frequency (CF). This metric is used to specifically validate the explicit enhancement capability of the FAM frequency-aware modulation module proposed in this paper for high-frequency textures:
  (20) $$SF=\sqrt{RF^{2}+CF^{2}}$$
  Here, RF and CF represent the root mean squares of gray-level differences between adjacent pixels in the horizontal and vertical directions, respectively.
  (21) $$RF=\sqrt{\frac{1}{H\left(W-1\right)}\sum_{i=1}^{H}\sum_{j=2}^{W}{\left(I_{fus}\left(i,j\right)-I_{fus}\left(i,j-1\right)\right)}^{2}}$$
  (22) $$CF=\sqrt{\frac{1}{\left(H-1\right)W}\sum_{i=2}^{H}\sum_{j=1}^{W}{\left(I_{fus}\left(i,j\right)-I_{fus}\left(i-1,j\right)\right)}^{2}}$$
5. Mutual Information (MI): Based on information theory, MI quantifies the total amount of information acquired and retained by the fused image from the two source images, reflecting the fidelity of feature transfer. Its calculation formula is defined as
  (23) $$MI=\sum_{a,f}P_{FA}\left(f,a\right)\log_{2}\frac{P_{FA}\left(f,a\right)}{P_{F}\left(f\right)P_{A}\left(a\right)}+\sum_{b,f}P_{FB}\left(f,b\right)\log_{2}\frac{P_{FB}\left(f,b\right)}{P_{F}\left(f\right)P_{B}\left(b\right)}$$
  where $P_{FA}\left(f,a\right)$ and $P_{FB}\left(f,b\right)$ denote the joint probability density distributions of source images A and B with the fused image F, respectively, while $P_{F}\left(f\right)$, $P_{A}\left(a\right)$ and $P_{B}\left(b\right)$ represent the corresponding marginal probability densities of each image.
6. The sum of correlations of differences (SCD) evaluates the correlation between the fused image and the difference maps of the source images; this metric reflects, to a certain extent, the degree to which the fused image incorporates complementary information from the source images:
  (24) $$SCD=r\left(I_{vi}^{Y},I_{fus}-I_{ir}\right)+r\left(I_{ir},I_{fus}-I_{vi}^{Y}\right)$$
  Here, r(⋅,⋅) denotes the Pearson correlation coefficient between two matrices, $I_{vi}^{Y}$ and $I_{ir}$ represent the visible luminance image and the infrared image, respectively.
7. Visual information fidelity (VIF) is an evaluation metric based on natural scene statistics and the human visual system. It estimates local statistics through multi-scale Gaussian filtering and, in combination with the natural scene statistics model, measures the degree of information fidelity of the fused image in the human visual channel. The specific calculation formula is as follows:
  (25) $$VIF\left(x,f\right)=\frac{\sum_{j=1}^{K}\sum_{i}\log_{10}\left(1+\frac{g_{j,i}^{2}\sigma_{x,j,i}^{2}}{\sigma_{v,j,i}^{2}+\sigma_{n}^{2}}\right)}{\sum_{j=1}^{K}\sum_{i}\log_{10}\left(1+\frac{\sigma_{x,j,i}^{2}}{\sigma_{n}^{2}}\right)}$$
  The final VIF score is obtained by summing the VIF values for the visible luminance image and the infrared image:
  (26) $$VIF=VIF\left(I_{vi}^{Y},I_{fus}\right)+VIF\left(I_{ir},I_{fus}\right)$$
8. Visual Edge Information Transfer Factor (Qabf, denoted as $Q^{AB/F}$ in equations) objectively evaluates the degree of retention of edge and contour details from the source images in the fused image, measuring the transmission loss of edge structural information by extracting the gradient magnitude and direction. Its calculation formula is defined as
  (27) $$Q^{AB/F}=\frac{\sum_{i=1}^{H}\sum_{j=1}^{W}\left(Q_{AF}\left(i,j\right)w_{A}\left(i,j\right)+Q_{BF}\left(i,j\right)w_{B}\left(i,j\right)\right)}{\sum_{i=1}^{H}\sum_{j=1}^{W}\left(w_{A}\left(i,j\right)+w_{B}\left(i,j\right)\right)}$$
  where $Q_{AF}\left(i,j\right)$ and $Q_{BF}\left(i,j\right)$ denote the edge information preservation degrees of source images A and B with respect to the fused image F at pixel (i,j), respectively, while $w_{A}\left(i,j\right)$ and $w_{B}\left(i,j\right)$ represent the gradient magnitude weights of source images A and B calculated at that point based on the Sobel operator.

### 4.3. Qualitative Visual Evaluation

#### 4.3.1. Visual Comparison on Benchmark Datasets

To comprehensively and rigorously evaluate the cross-domain generalization capability and visual robustness of the fusion model under complex real-world physical degradation conditions, this paper conducts systematic qualitative comparisons on four highly challenging benchmark datasets. To visually examine the reconstruction capability of each compared network for high-frequency topological structures and low-level features, red boxes are uniformly used to provide localized close-up comparisons of key regions where feature degradation is most likely to occur.

**Figure 4:**
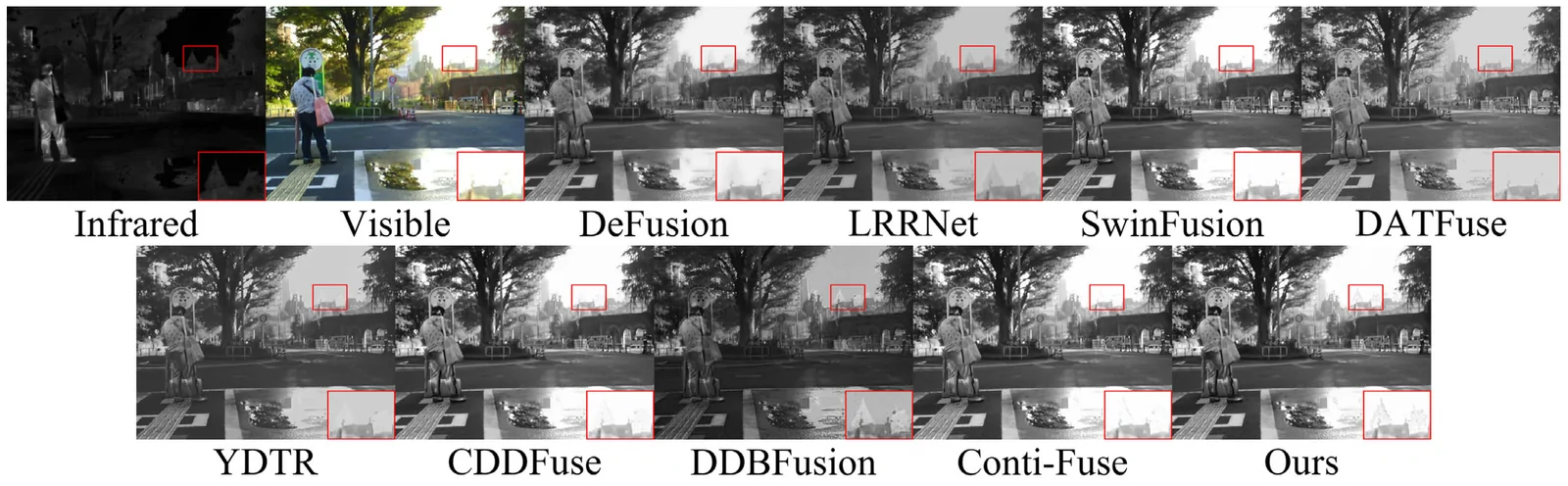
Localized comparison on the MSRS dataset (The red box denotes the locally enlarged region for detail illustration).

MSRS Complex Urban Scene Visual Comparison: The MSRS dataset contains highly challenging complex urban scenes. As shown in Figure 4, when the visible modality suffers from strong light scattering, leading to degradation in distant scenes, some comparison methods suffer from global brightness loss, resulting in reduced perceptual realism. Meanwhile, SwinFusion, DeFusion, and recent DDBFusion all exhibit significant oversmoothing when processing high-frequency details in distant scenes. In contrast, HL-Fuse preserves the brightness response of infrared targets relatively well in this scene while maintaining part of the visible background texture. Within the red-box region, building edges and roof structures remain relatively clear, indicating that the proposed method has good structural preservation capability in this example. This also suggests its potential applicability to urban remote sensing interpretation and UAV visual perception.

**Figure 5:**
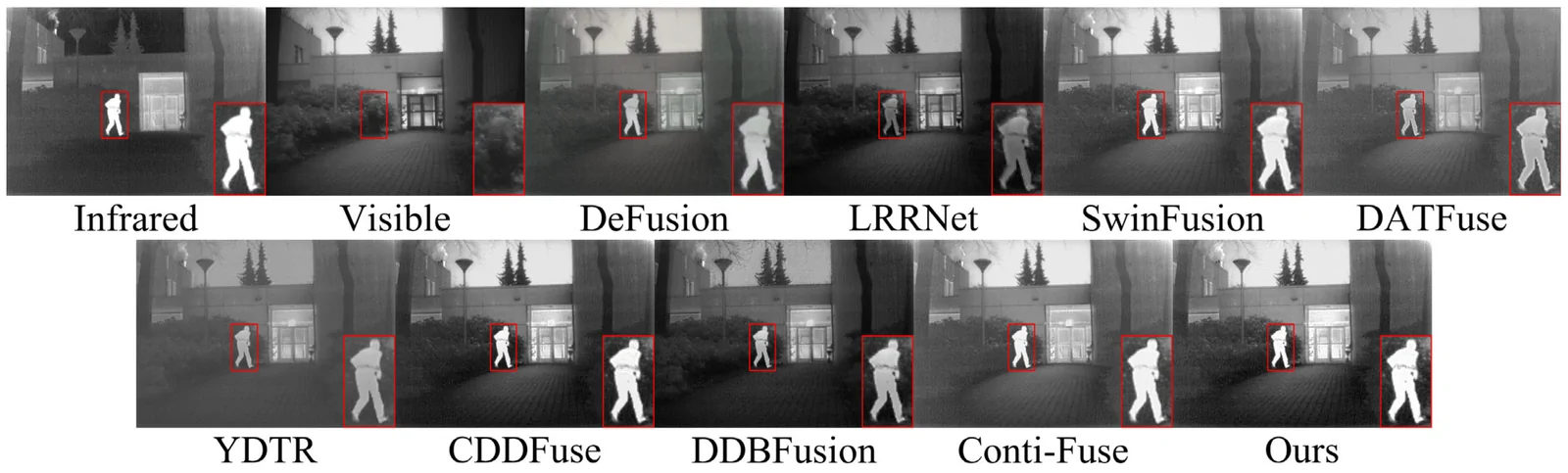
Localized comparison on the TNO dataset (The red box denotes the locally enlarged region for detail illustration).

Visual Comparison of TNO Nighttime Extreme Occlusion Scenarios: The TNO dataset focuses on low-illumination and low-light scenarios, aiming to evaluate the ability of different models to highlight and identify occluded thermal targets under severe visual masking conditions. As shown in Figure 5, methods such as SwinFusion and DATFuse are observed to produce significant feature neutralization during fusion, causing the pedestrian within the red box to exhibit “translucent artifacts” with extremely low contrast. Although CDDFuse maintains the structure relatively well, it remains conservative in terms of edge separation. In contrast, HL-Fuse maintains better contrast for the pedestrian thermal target within the red-box region, with a more pronounced grayscale difference between the target contour and the background. This helps improve the distinguishability of target regions in low-light scenarios, demonstrating its certain reference value for applications such as nighttime remote-sensing inspection and security surveillance.

**Figure 6:**
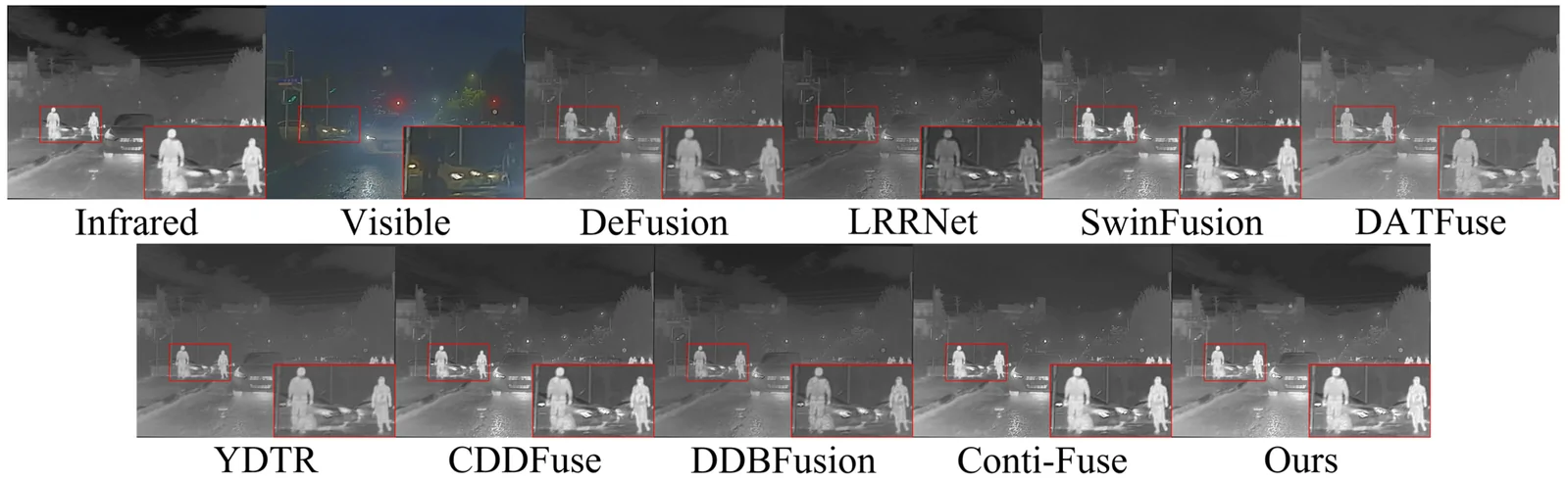
Localized comparison on the M^3^FD dataset (The red box denotes the locally enlarged region for detail illustration).

Visual Comparison of M^3^FD High-Dynamic-Range Illumination Scenes: Faced with the demanding challenges of the M^3^FD dataset, where intense glare coexists with extreme darkness, as shown in Figure 6, DeFusion introduces high-frequency noise and causes distortion in reflections from accumulated water when handling complex lighting scenarios such as waterlogged roads. In contrast, for baseline methods such as YDTR and LRRNet, infrared thermal targets are severely diluted by large areas of low-frequency dark regions, resulting in dim pedestrians with blurred boundaries within the red box. The HL-Fuse method proposed in this paper addresses this high-dynamic-range challenge. The model effectively suppresses the feature suppression of salient targets caused by dark regions and glare in the visible image. This not only significantly enhances the thermal saliency of the pedestrian within the red box, but also largely preserves the high-frequency details of the roadside sign pole nearby, thereby improving the semantic distinguishability of nighttime scenes and providing relatively stable visual input for UAV nighttime observation and high-dynamic-range remote sensing scenarios.

**Figure 7:**
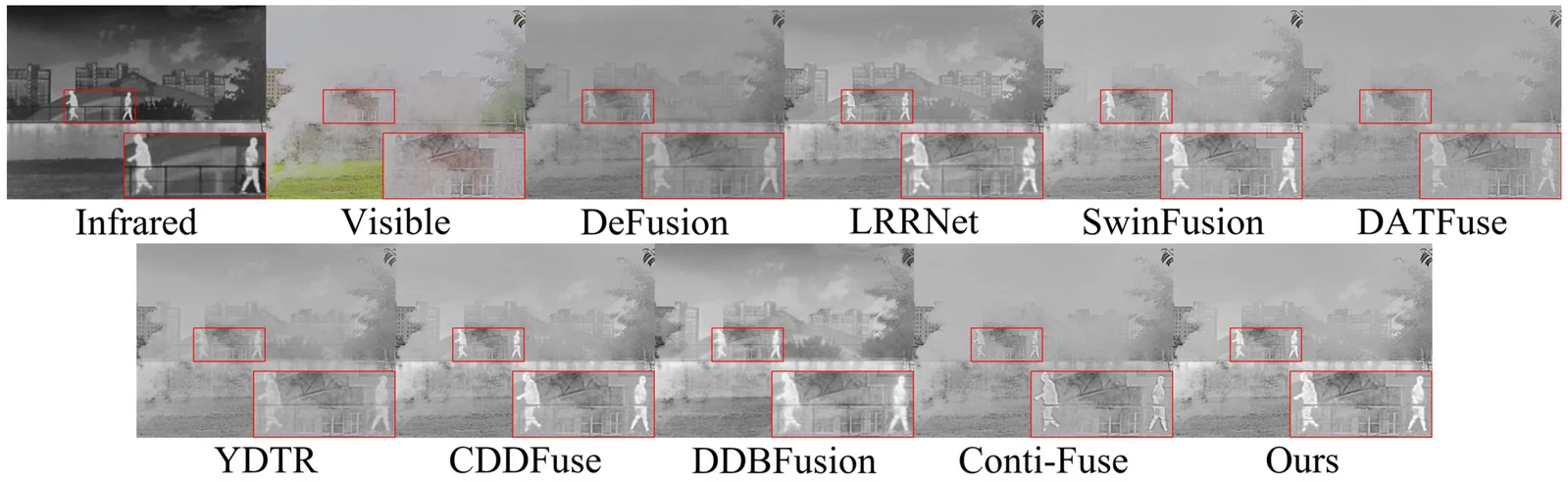
Localized comparison on the FMB dataset (The red box denotes the locally enlarged region for detail illustration).

Visual Comparison of FMB Extreme Dense-Fog Scattering Scenarios: In the FMB dense-fog degradation environment, as shown in Figure 7, many compared methods suffer from “feature dilution” due to severe atmospheric scattering: infrared thermal targets are neutralized by dense fog, resulting in a significant loss of saliency for the pedestrian within the red box, while the foreground railing appears noticeably oversmoothed and almost blends into the fog. In contrast, HL-Fuse demonstrates stable high-frequency feature anchoring capability. It maintains good saliency for the pedestrian thermal target, and linear structures such as the railing are clearer than those produced by some compared methods. This indicates that the proposed method still has a certain degree of cross-scene generalization potential under degraded conditions such as foggy weather.

#### 4.3.2. Subjective Visual Evaluation

To further validate the image quality from the perspective of human visual perception, this study conducted a subjective visual evaluation building upon the qualitative visual comparisons. A total of 33 valid questionnaires were collected for this survey. The participants’ familiarity with the fields of image processing and computer vision is distributed as follows: 30.30% have relevant learning or research experience, 24.24% have some understanding, and 45.45% have no professional background in these areas. Regarding visual health, 90.91% of the participants reported normal or corrected-to-normal vision, and 90.91% reported no color vision deficiencies. In terms of the selection of comparison baselines, three representative advanced algorithms—SwinFusion, CDDFuse, and Conti-Fuse—were introduced. These models respectively represent the core technological paradigms of Transformer-based global perception, dual-branch feature decoupling, and continuous manifold representation in the current field of multimodal image fusion, thereby providing comprehensive and fair benchmarks to evaluate the standing of the proposed method. The evaluation was conducted using a blind ranking approach across scenes from four datasets: MSRS, TNO, M^3^FD and FMB. Without knowing the names of the algorithms, observers ranked the fusion results of the four methods in descending order of preference based on the salience of thermal targets, the clarity of visible background textures, and the overall naturalness of the images. The comprehensive scores were calculated based on an average weighted rule derived from the ranking results, where scores from 4 to 1 were assigned from the highest to the lowest preference levels, with a maximum score of 4. A higher score indicates superior subjective visual acceptability.

The statistical results of the subjective comprehensive scores for the different algorithms are presented in Table 1. In terms of the specific performance across individual datasets, the proposed method achieved subjective scores of 2.82, 2.91 and 2.73 on the MSRS, TNO and FMB datasets, respectively, outperforming the other three comparison algorithms. This outcome indicates that the proposed method offers superior visual acceptability in terms of highlighting thermal targets and preserving background details. On the high-dynamic-range M^3^FD dataset, CDDFuse and SwinFusion obtained subjective scores of 2.73 and 2.72, respectively, which are slightly higher than the 2.52 achieved by the proposed method. Despite this, looking at the comprehensive evaluation across all four datasets, the proposed method still demonstrates the most stable overall generalization performance during cross-scene testing. In contrast, the scores of the Conti-Fuse algorithm across the datasets range from 1.64 to 1.97, reflecting a relatively lower overall visual perception rating. In summary, the statistical results of the subjective user study validate the effectiveness of the proposed method in optimizing the human visual perception experience.

### 4.4. Quantitative Evaluation

**Figure 8:**
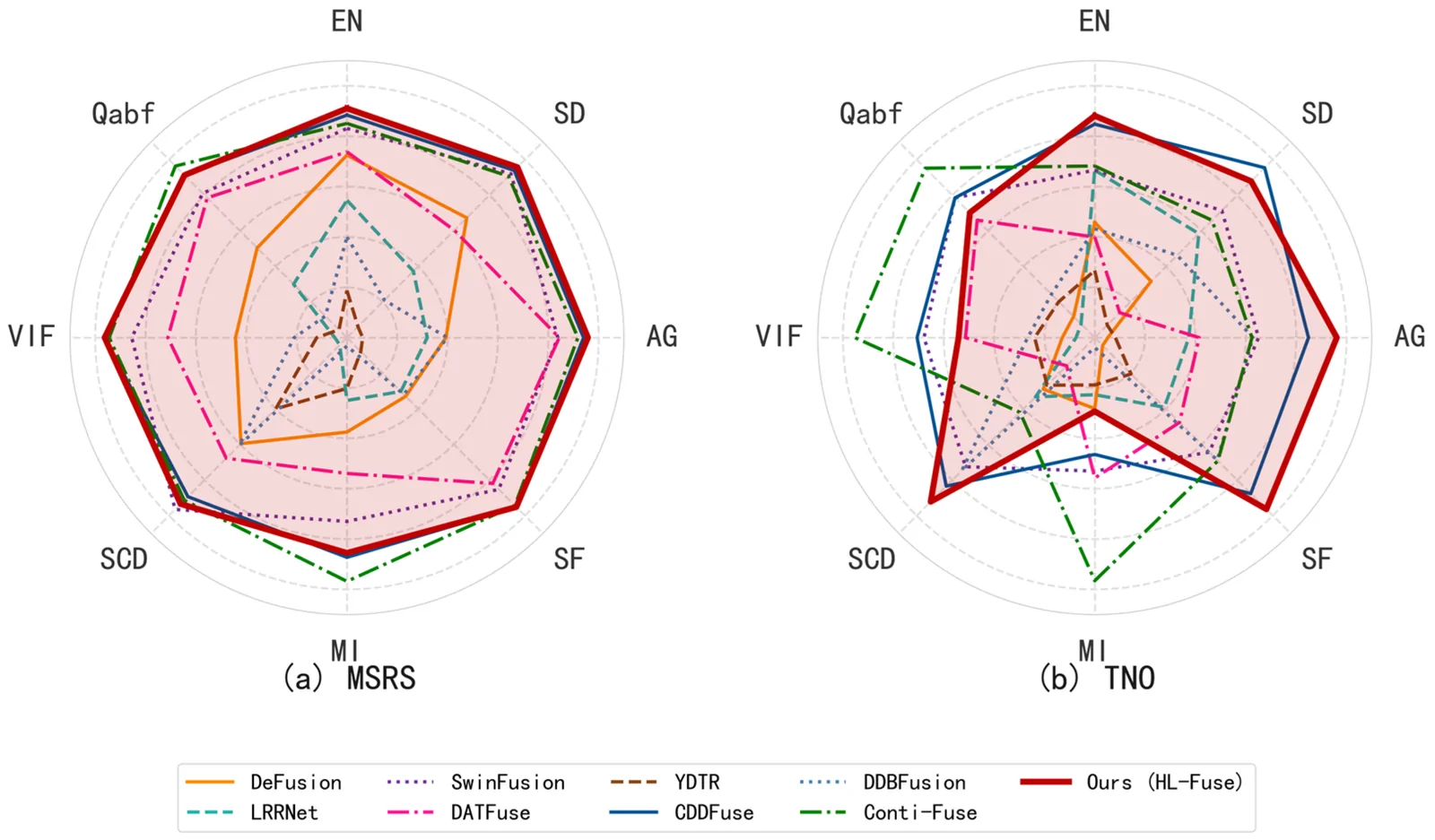
Multidimensional quantitative evaluation radar charts of different fusion methods on the (**a**) MSRS and (**b**) TNO datasets.

Structural Fidelity and Global Contrast Analysis (MSRS and TNO Datasets): As shown in Table 2 and Figure 8a, in the complex urban scenes of the MSRS dataset, the proposed HL-Fuse attempts to strike a better balance between thermal target enhancement and detail preservation. From the objective evaluation, the model yields relatively leading scores on metrics such as EN, SD, AG, and SF, while maintaining favorable competitiveness in SCD, VIF, and Qabf. This result suggests the model’s potential in enhancing global contrast, enriching image information, and preserving edge details.

Furthermore, when addressing the challenge of target enhancement in extremely dark regions of the TNO nighttime dataset, as shown in Table 3 and Figure 8b, HL-Fuse also demonstrates positive adaptability on EN, SF, AG and SCD. Objectively speaking, although the model does not yield the highest scores on metrics like SD, VIF, or Qabf in certain scenarios, it can be observed from the radar charts that its overall distribution across all indicators is relatively balanced. These quantitative results indicate that rather than focusing on the extreme maximization of a single metric, the design of HL-Fuse leans towards seeking a reasonable compromise between complementary information incorporation and visual structural stability, aiming to mitigate the risk of visual imbalance caused by single-metric-driven optimization. This feature reconstruction paradigm, characterized by strategic trade-offs, aligns to some extent with the practical requirements of prioritizing high-value target discovery in complex monitoring scenarios.

**Figure 9:**
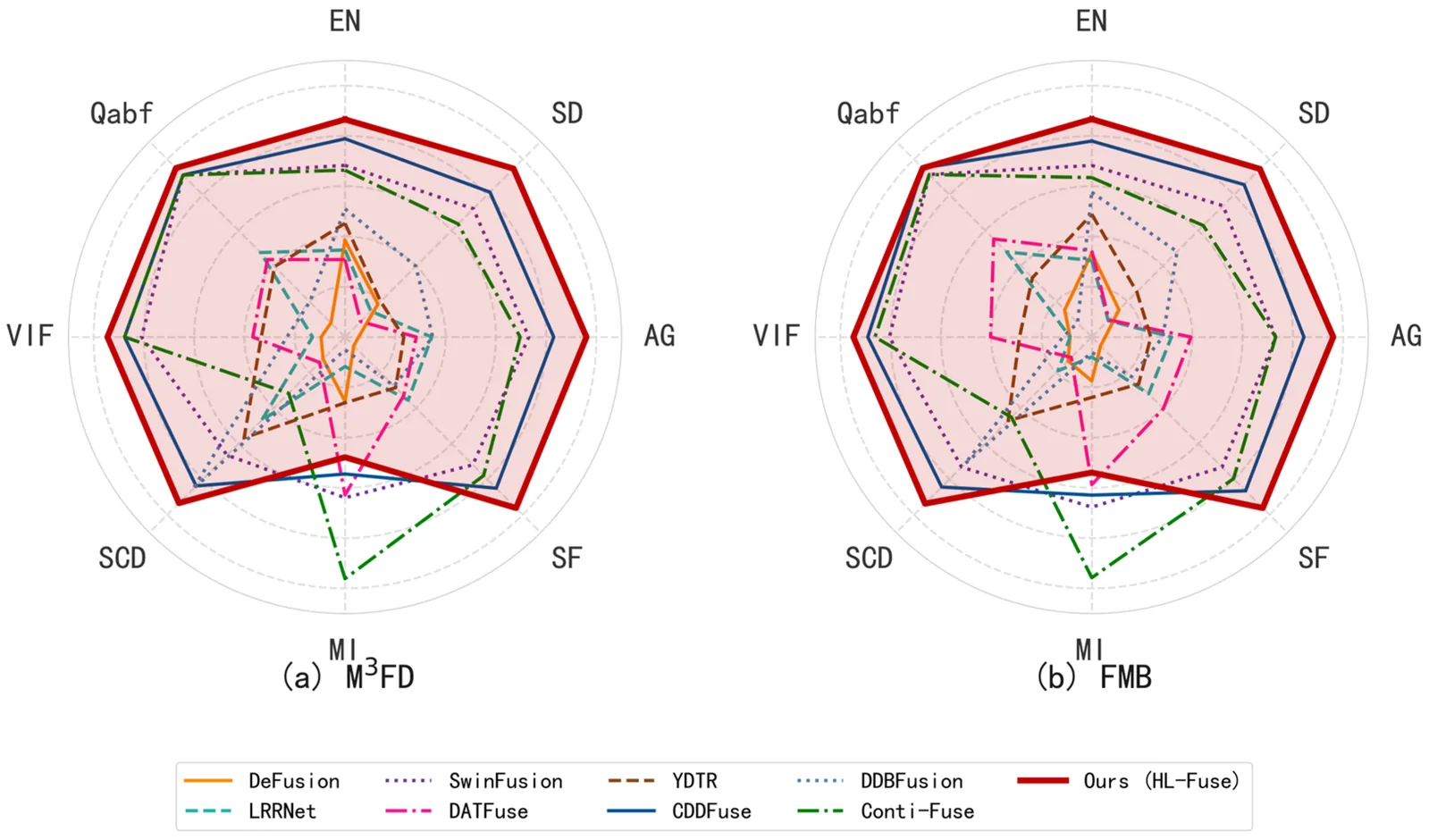
Radar chart showing multidimensional quantitative evaluation of different fusion methods on the (**a**) M3FD and (**b**) FMB datasets.

Robustness Analysis in Complex Degraded Scenarios (M^3^FD and FMB Datasets): When faced with high-dynamic and non-stationary light-source interference in the M^3^FD dataset, as shown in Table 4 and Figure 9a, the proposed HL-Fuse achieved competitive performance on most of the objective metrics (e.g., VIF, SD and SCD), though not universally superior. Some compared methods performed better on the Mutual Information (MI) and Qabf metrics, but their visual results occasionally exhibited glare diffusion, unnatural edge over-enhancement, or local texture distortion. This observation suggests that optimization on a single metric does not necessarily translate to superior subjective visual quality. In contrast, HL-Fuse tended to provide a relatively balanced trade-off between visual information preservation and global contrast, which is beneficial for such challenging scenes in a practical sense. Similarly, in scenes with physical occlusion caused by dense fog in the FMB dataset, as shown in Table 5 and Figure 9b, HL-Fuse maintained reasonably high scores across almost all evaluation metrics (e.g., SCD and VIF), with the exception of MI and Qabf. Some compared methods showed noticeable modal bias or artificial sharpening when handling complex environments, leading to a decline in key metrics (e.g., VIF) and the generation of localized blackout artifacts. HL-Fuse generally preserved details effectively by providing critical high-frequency anchoring, and its distribution in the radar charts exhibited a relatively balanced and comprehensive pattern. This implies that the model does not excessively absorb interference artifacts such as glare or dense fog, but rather tends to reflect valid scene information in a reasonably objective manner. This overall performance shows promise for potential application in all-weather remote sensing and Earth observation tasks, although further validation under more diverse conditions would be desirable.

### 4.5. Ablation Study and Parameter Sensitivity Analysis

To comprehensively evaluate the structural rationality and hyperparameter robustness of the proposed model (HL-Fuse), this section conducts systematic ablation studies on core modules and sensitivity analyses on critical parameters. First, by progressively stripping or replacing the core components within the network, the individual contributions of separate modules during feature reconstruction and complementary information incorporation are quantitatively measured. Subsequently, by varying the values of critical parameters, the changes in individual testing metrics are observed to evaluate the model’s stability under different parameter configurations.

#### 4.5.1. Ablation Study on Core Modules

To validate the effectiveness and synergistic contributions of the core architecture of HL-Fuse, particularly FAM and the Hyper-LoRA, we conducted systematic structural ablation experiments on the MSRS dataset. As shown in the ablation matrix in Table 6, we used an architecture comprising only base feature decoupling and reconstruction as the baseline model (Baseline, $M_{1}$), and constructed multiple variants by sequentially introducing or replacing key components to objectively isolate the performance gains of each module.

Specifically, variant $M_{2}$ introduces FAM to independently validate the benefits of frequency-domain interaction for high-frequency feature extraction. Variants $M_{3}$ and $M_{4}$ introduce conventional static low-rank modulation and dynamic modulation, respectively, aiming to intuitively demonstrate the advantage of the dynamic environment-aware mechanism in handling complex lighting conditions. Variant $M_{5}$ combines FAM with Fixed-LoRA, representing a conventional hybrid approach lacking dynamic awareness. Ultimately, the complete model proposed in this paper (Ours) synergistically integrates FAM and the dynamic Hyper-LoRA mechanism on top of the baseline, achieving comprehensive improvements in both scene awareness and high-frequency extraction.

It should be particularly emphasized that, to adhere to a strict controlled-variable protocol, all variants maintain training parameters and joint loss constraints that are fully consistent with the final model, including the $L_{grad}$ and $L_{sd}$, among others. Furthermore, the base feature decoupling loss from the first stage is retained by default to ensure that the encoder does not experience feature collapse. This section aims to provide an in-depth analysis, purely from a structural perspective, of the sources of performance gains from the dynamic parameter mechanism and the frequency-domain modulation module.

**Figure 10:**
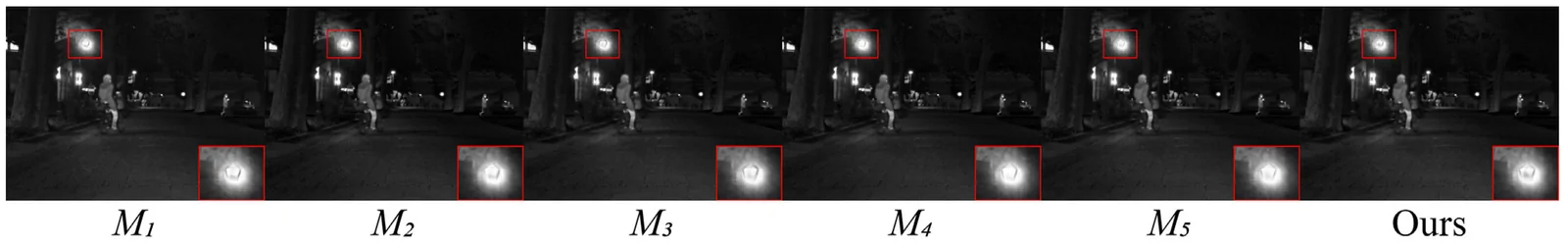
Comparison of zoomed-in results from ablation experiments on the MSRS dataset (The red box denotes the locally enlarged region for detail illustration).

From the qualitative visual results in Figure 10, in nighttime streetlight scenes, some variant models are susceptible to local high-brightness regions and background halos, resulting in glare diffusion or texture weakening. In contrast, the full HL-Fuse balances the suppression of high-brightness regions and the preservation of background textures, enabling it to retain thermally salient targets while maintaining local structural information.

Quantitative results are presented in Table 6. Notably, variant $M_{2}$, which solely introduces frequency-domain modulation, exhibits slightly lower AG and SF compared to the baseline ($M_{1}$). This phenomenon reveals that directly injecting high-frequency information without the guidance of dynamic environmental awareness can trigger local feature conflicts. Meanwhile, the metric fluctuations among the intermediate variants ($M_{3}$ to $M_{5}$) further indicate that the mere stacking of individual modules struggles to achieve a global optimum. However, this precisely demonstrates the strong interdependence among our proposed components.

**Figure 11:**
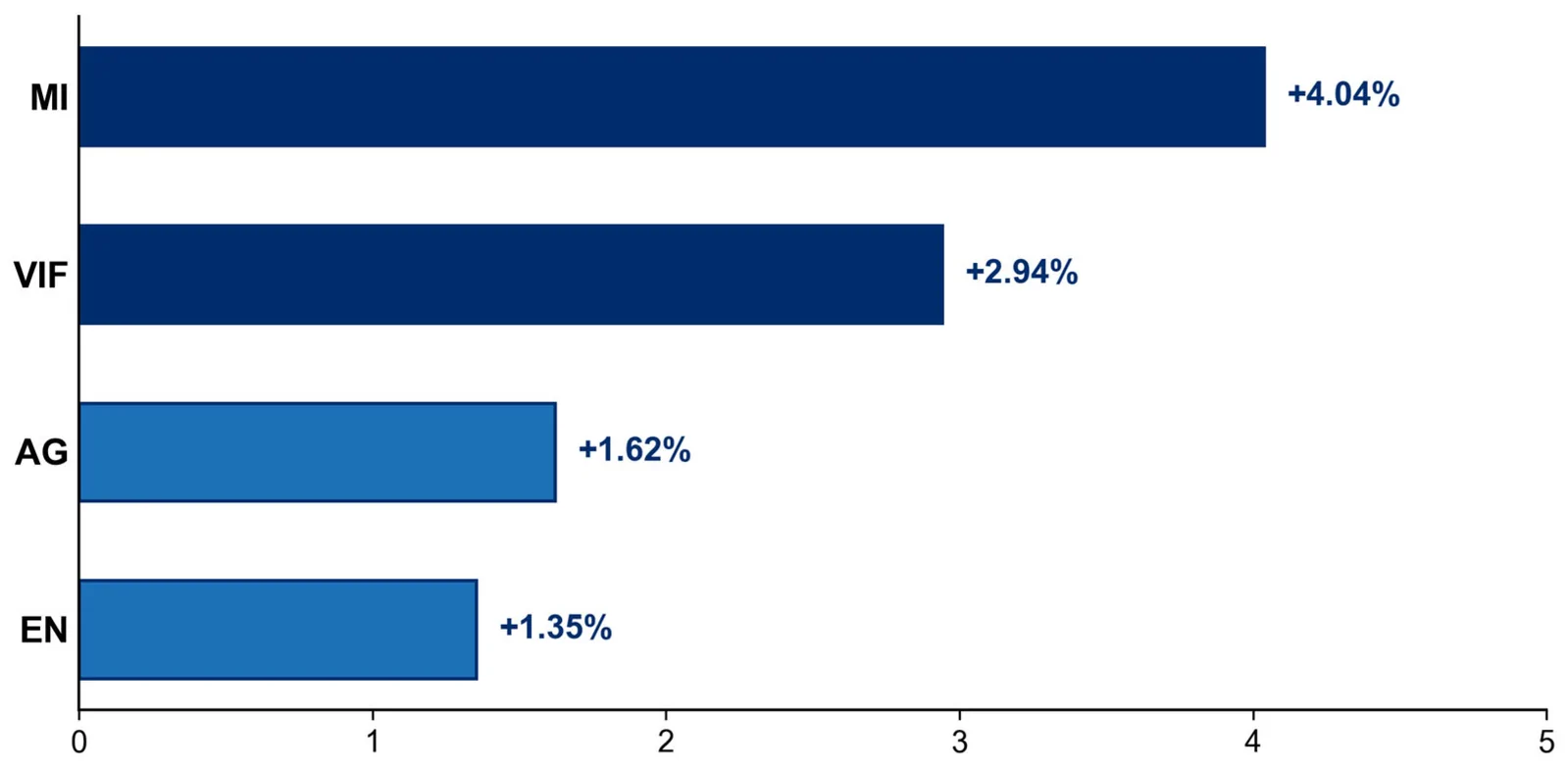
Percentage gains of our method over the baseline model.

As in Figure 11, when FAM is deeply coupled with the dynamic Hyper-LoRA mechanism in the complete model (Ours), the metrics achieve growth. Specifically, across four representative metrics, the complete model yields significant relative increases of 4.04% in MI, 2.94% in VIF, 1.62% in AG and 1.35% in EN over the baseline. Simultaneously, the remaining metrics (SD, SF, SCD and Qabf) also realize comprehensive synchronous improvements. Overall, rather than being a naive superposition of components, the complete HL-Fuse leverages effective mechanistic synergy to fundamentally overcome the local trade-offs caused by single-module introductions.

**Figure A1:**
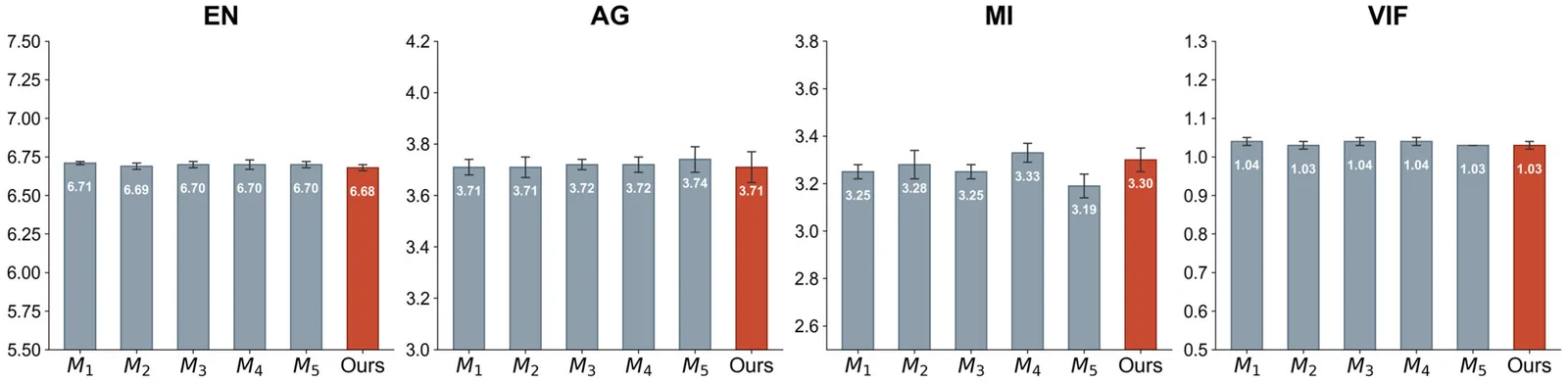
Performance Comparison of Ablation Models with Standard Deviation Error Bars.

Finally, to mitigate the potential fortuity introduced by random initialization, all variants were repeatedly trained and evaluated using three distinct random seeds. The detailed quantitative results across these multiple runs, including means and standard deviations, are provided in Appendix A (Table A2 and Figure A1). These analyses further confirm the parametric stability of the proposed architecture and the objectivity of the reported performance trends.

#### 4.5.2. Sensitivity Analysis

To validate the rationality of the core parameter configurations in the proposed method, we selected four core objective metrics, namely EN, AG, MI, and VIF, as the evaluation benchmarks to conduct a comprehensive sensitivity analysis on the dynamic low-rank modulation dimension and the frequency-domain cutoff scale.

**Figure 12:**
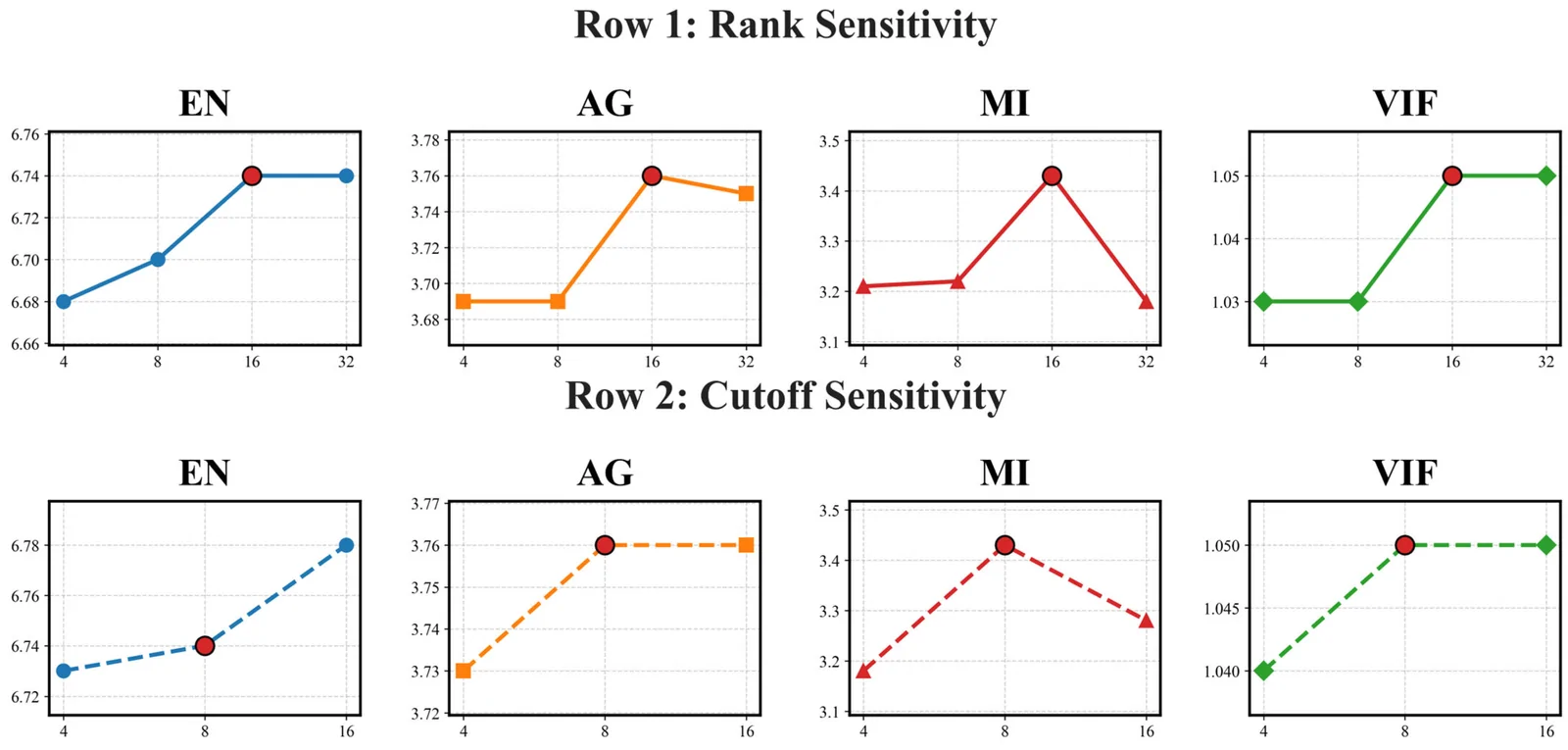
Parameter sensitivity analysis on the MSRS dataset for Rank and Cutoff (Red points indicate the selected parameter values).

As illustrated in Figure 12, we quantitatively present the specific trends of the aforementioned core metrics in response to hyperparameter variations. Regarding the dynamic low-rank modulation dimension, as shown in the first row of Figure 12, we evaluated the model’s performance when this hyperparameter was set to 4, 8, 16 and 32. Experimental results indicate that as the dimension increases from 4 to 16, the fusion performance across the four core metrics exhibits an overall upward trend, reaching their optimal or saturated states at a dimension of 16. This suggests that moderately expanding the low-rank subspace capacity improves the capability of the scene hypernetwork to capture environmental degradations and represent cross-modal features. However, when the dimension is further increased to 32, the performance gains for EN and VIF completely flatten out, whereas AG and MI exhibit a distinct decline. Consequently, the dynamic low-rank modulation dimension is set to 16 to achieve a balance between fusion performance and computational overhead. Regarding the frequency-domain cutoff denominator, as shown in the second row of Figure 12, we evaluated the model’s performance when this hyperparameter was set to 4, 8 and 16. The results demonstrate that AG, MI, and VIF attain their maximum values when the denominator is set to 8. Further increasing the denominator to 16 yields a slight gain in EN but causes a decrease in the remaining metrics due to the excessive compression of large-scale low-frequency structures. Therefore, the value of 8 is selected as the default frequency-domain cutoff denominator to achieve a balance between maintaining global structural integrity and enhancing edge texture details.

Furthermore, to validate the computational efficiency of the proposed hyper-modulation mechanism, comprehensive benchmarking was conducted on various low-rank dimension variants. The evaluation was performed on a single NVIDIA RTX 5060 GPU at a baseline image resolution of 128×128, focusing on five core metrics: parameter count, total computational complexity (FLOPs), peak Video Random Access Memory (VRAM) usage, inference time, and frames per second (FPS). The detailed results are presented in Table 7. The quantitative data indicate that despite the continuous increase in the dynamic low-rank modulation dimension, the total FLOPs of the entire fusion network consistently remain stable at approximately 29.22 G. Meanwhile, as the dimension increases from 4 to 32, the total parameter count and dynamic peak VRAM consumption exhibit only marginal growth, while the average inference time remains highly stable (fluctuating slightly around 22–24 ms). Under the optimal configuration with the dynamic low-rank modulation dimension set to 16, the model achieves a real-time processing speed of 44.31 FPS while maintaining low computational resource consumption. These quantitative experimental results demonstrate that the execution of the scene hypernetwork module does not introduce any significant additional computational burden to the backbone network, confirming that the proposed model achieves an ideal balance between fusion quality and computational efficiency.

### 4.6. Scene-Adaptive Analysis

In practical infrared and visible image fusion applications, variations in environmental lighting conditions (such as day-and-night alternation) affect the feature distributions of the images. Traditional fusion networks with static weights often struggle to simultaneously accommodate feature extraction across different lighting scenarios. To verify the feature extraction capability of the proposed model under varying illumination conditions, we utilized the lighting labels provided by the MSRS dataset to divide the test set into daytime and nighttime subsets, and conducted quantitative comparisons respectively.

Table 8 and Table 9 present the objective evaluation metrics of the compared methods in daytime and nighttime scenarios, respectively. In daytime scenes, visible images typically contain rich background texture information. The experimental results indicate that the proposed method achieves the highest values across four core metrics: EN, SD, AG and SF. This demonstrates that under sufficient lighting conditions, the model can effectively extract high-frequency texture details while maintaining good image contrast and spatial clarity. In nighttime scenes, the quality of visible images suffers degradation, making the fusion task rely more heavily on the thermal radiation features provided by infrared images. As shown in Table 9, the proposed method also achieves the highest scores in metrics including EN, SD, SCD and VIF. These results indicate that in low-illumination environments, the model can effectively transfer the correlation features between source images while maintaining image fidelity that conforms to human visual perception.

**Figure 13:**
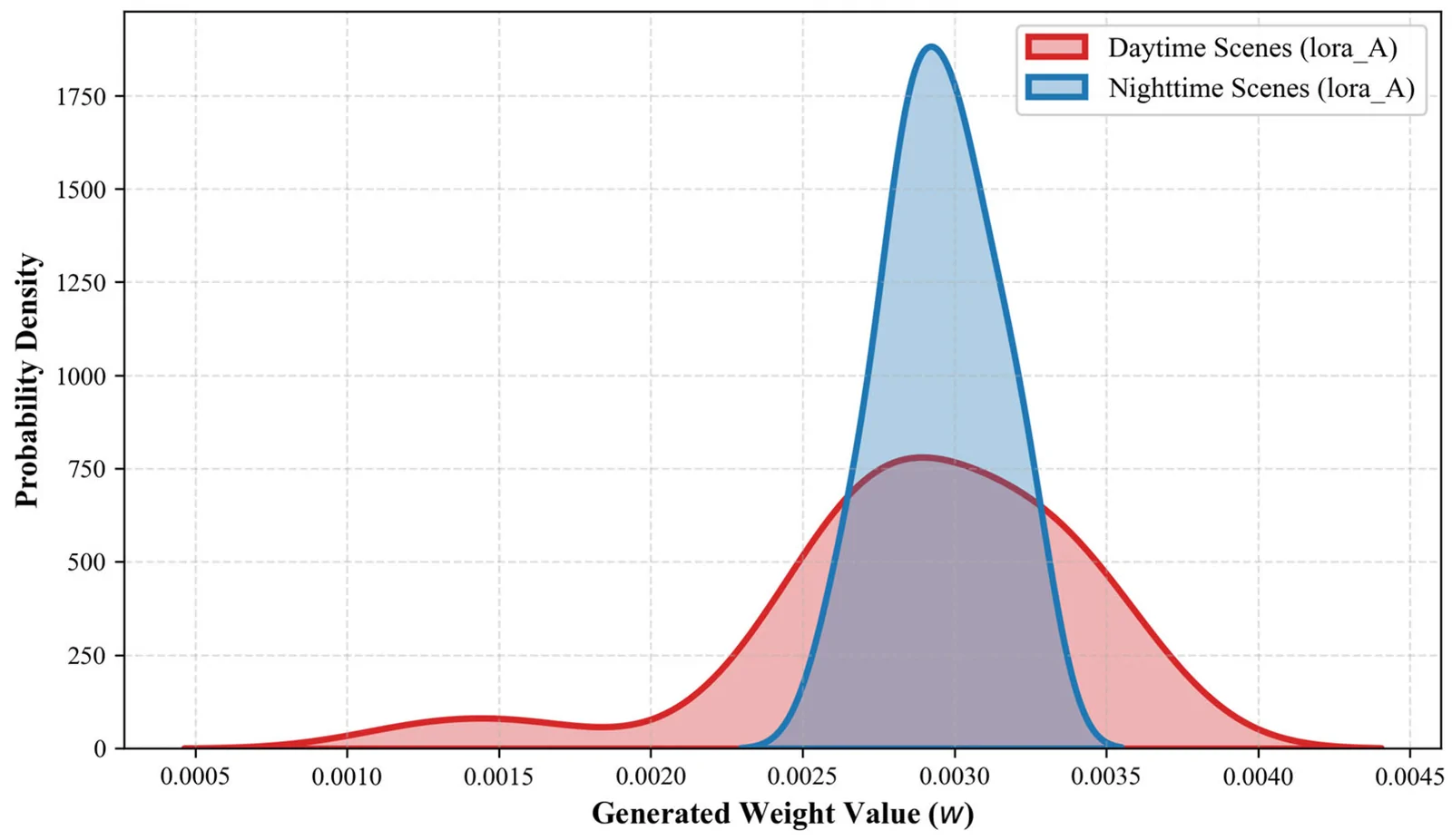
1D density distribution of Hyper-LoRA weights for daytime and nighttime scenes.

To further illustrate the response mechanism of the internal model weights to scene variations, Figure 13 plots the 1D probability density distribution curves of the low-rank modulation parameters (represented by lora_A) generated by SceneHyperNet under daytime and nighttime scenarios. It can be observed that the generated parameter distributions exhibit distinct differences under varying illumination conditions. In the daytime scenario (red curve), the parameter distribution has a larger variance, spans a wider range, and presents a secondary peak in the lower-value region. This indicates that due to the rich and diverse features of daytime visible scenes, the modulation weights generated by the hypernetwork possess higher diversity and dynamic adjustment space. Conversely, in the nighttime scenario (blue curve), the parameter distribution shrinks significantly, presenting a sharp unimodal shape and is entirely concentrated in the high probability density region. This suggests that in low-illumination environments, with the reduction in effective visible information, the hypernetwork tends to output more compact and consistent modulation parameters to stabilize the extraction of infrared targets.

In summary, compared to the baseline methods, the proposed model exhibits a relatively balanced performance across different lighting conditions. This cross-scene stability primarily stems from the designed SceneHyperNet dynamic modulation mechanism. By extracting environmental degradation priors, the model can adaptively generate low-rank modulation weights, leaning towards visible texture preservation during the day and prominent infrared target extraction at night, thereby achieving adaptive fusion for complex illumination scenes.

### 4.7. Downstream Tasks

To further evaluate the impact of fused images on downstream visual tasks, this paper adopts object detection as a cascaded validation task and evaluates the fused images generated by each comparison method. The experiments are conducted on the official split of the MSRS dataset and focus on the two core categories, namely “person” and “car”, which are of particular importance for autonomous driving and urban security applications. The detection evaluation set is constructed using the publicly available MSRS detection annotations [46]. Specifically, we use a lightweight YOLOv5s model initialized with weights pre-trained on the MS-COCO dataset as the downstream object detector. For each fusion method, the detector is independently trained using the 1083 fused images in the training set generated by that method under the same hyperparameter configuration. During training, the maximum number of epochs is set to 100, and an early stopping strategy with a patience of 20 epochs is adopted to obtain stable detector weights. The resulting optimal weights are then used to perform inference on the 361 fused test images generated by the corresponding fusion method. This evaluation protocol provides a task-level assessment of how object structures, edge information, and potential artifacts in different fusion results affect downstream object detection performance.

**Figure 14:**
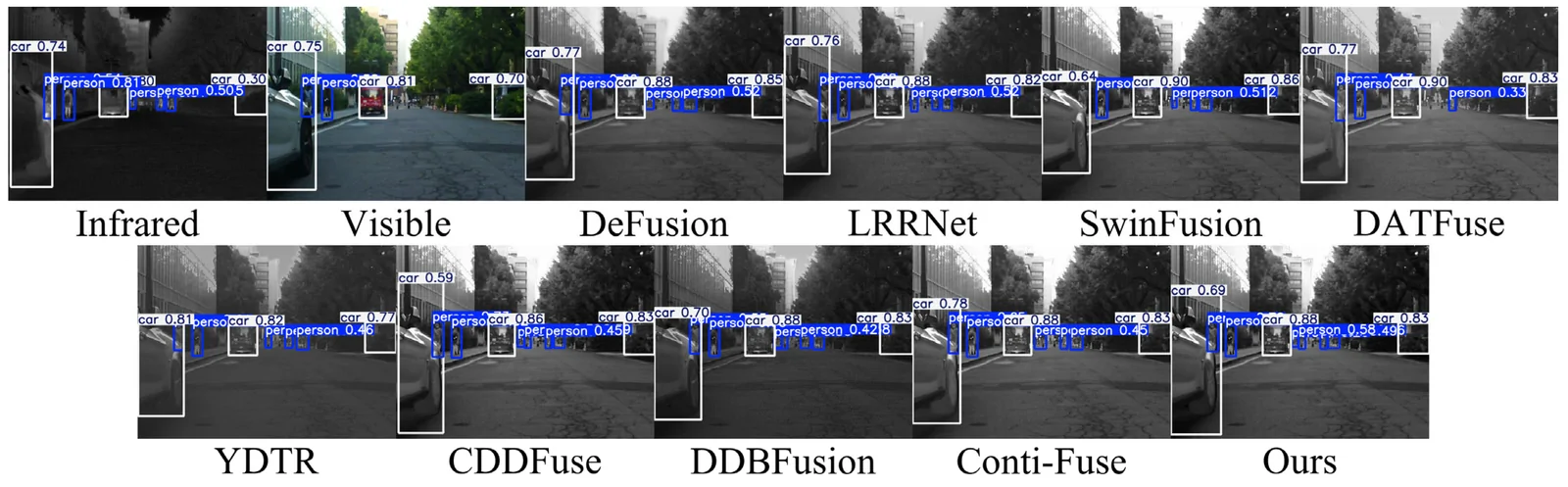
Visual Comparison of Object Detection.

Regarding quantitative evaluation metrics, we adopt mean average precision (mAP) as the core metric. The conventional mAP@0.5 reflects the overall recall capability and detection lower bound of the detector driven by the fused images, whereas mAP@0.5:0.95, which incorporates a continuous range of Intersection-over-Union thresholds from 0.5 to 0.95, imposes extremely stringent pixel-level requirements on the localization accuracy of target bounding boxes. A higher mAP@0.5:0.95 typically indicates more accurate detection box localization and can therefore be used to further evaluate the impact of object boundaries and structural information in the fused images on the detection task. The quantitative detection results driven by each fusion method are summarized in Table 10, and the visual comparison of detection bounding boxes in typical complex scenes is shown in Figure 14.

To further evaluate the impact of fused images on high-level visual tasks, this paper feeds the fused images generated by different methods into a pretrained YOLOv5s detector and evaluates them for object detection on the MSRS dataset. Downstream detection experiments provide additional insight at the task level into whether the fusion results help preserve object-related structural and semantic information.

As shown in the visualization results in Figure 14, the impact of different fusion methods on detection results varies to some extent. Infrared images highlight thermally salient targets such as pedestrians, while visible images capture the textures and geometric structures of vehicles, roads, and the background street scene. Some methods exhibit brightness attenuation or localized texture blurring during the fusion process, which may affect the detector’s ability to identify distant small objects and low-contrast targets. In contrast, HL-Fuse preserves the thermal saliency of pedestrian regions relatively well in this example while retaining structural information from vehicles and the background, resulting in relatively stable detection performance for foreground objects and some distant small-scale targets. Quantitative results are shown in Table 10. In the “person” category, HL-Fuse achieved an mAP@0.5 of 0.851 and an mAP@0.5:0.95 of 0.487, both of which were the highest values among the compared methods, indicating that the fused images generated by our method have a positive effect on pedestrian detection. This may be attributed to the fact that the fusion results effectively preserve the thermal saliency and local boundary structures of pedestrians. For the composite “all” metric, HL-Fuse achieved an mAP@0.5 of 0.849 and an mAP@0.5:0.95 of 0.523, both of which were the best results and showed an evident improvement. This indicates that HL-Fuse has a certain advantage in overall detection performance. However, the magnitude of this improvement is relatively limited; therefore, this study treats it as supporting evidence that the fusion results offer potential benefits for downstream detection tasks.

It should be noted that HL-Fuse did not achieve the highest results for the “car” category. SwinFusion achieved the highest values for both “car” mAP@0.5 and mAP@0.5:0.95, while the corresponding results of HL-Fuse were 0.846 and 0.558, which are close to the optimal levels. This indicates that our method remains competitive in vehicle detection, but different object categories have varying requirements for fused images. Vehicle objects typically rely more on geometric contours and local textures in visible images; during the fusion of grayscale input and infrared information, some vehicle appearance details may be compromised. This phenomenon suggests that how to better preserve the details of visible structural objects, such as vehicles, while enhancing thermally salient targets remains a topic for further research.

Overall, HL-Fuse achieved good comprehensive performance in the MSRS downstream object detection experiments, outperforming the compared methods particularly in the “person” category and the composite “all” metric. This indicates that the fused images generated by our method not only possess a certain level of visual quality, but also provide relatively effective input for object detection tasks. However, the detection gain is not entirely consistent across different classes. Further validation of its generalization ability in complex remote sensing scenarios is still needed by incorporating more detectors, additional datasets, and task-driven training strategies.

## 5. Discussion

In complex urban environments, image fusion systems are often affected by degradation factors such as dense fog scattering, strong light interference, and low illumination. Most static fusion frameworks lack dynamic adaptation capabilities, making them prone to incorporating contaminated modal features. In contrast, HL-Fuse performs adaptive calibration of the base feature fusion process through scene-prior-driven dynamic low-rank modulation, achieving a favorable balance between preserving thermally salient targets and enhancing edge details.

Although HL-Fuse exhibits strong robustness, certain limitations remain for real-world deployment. First, under extreme non-stationary lighting conditions (e.g., direct headlights combined with specular reflections from standing water), the fused results may still suffer from local attenuation of high-frequency details or halo artifacts. Furthermore, for extremely small targets hidden in cluttered backgrounds, the enhancement of fine-grained boundaries remains insufficient, indicating the need for more fine-grained target-aware mechanisms in the future.

Second, this study is based on the assumption that ideal spatial registration of the source images has been completed. However, in practical in-vehicle perception applications, heterogeneous sensors inevitably introduce minute pixel-level parallax and jitter. To explore the robustness boundary of the model against geometric perturbations, we conducted a preliminary spatial perturbation test by artificially introducing a 3-pixel spatial translation.

**Figure 15:**
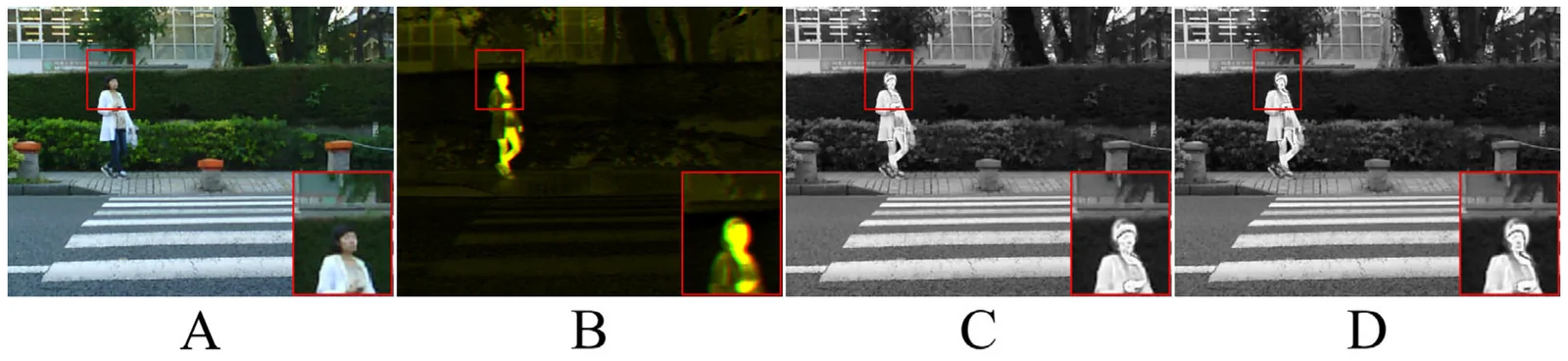
Visual comparison of fusion robustness under a 3-pixel spatial shift on the MSRS dataset ((**A**): visible image; (**B**): misalignment overlay with red for IR and green for VIS; (**C**): ours with aligned input; (**D**): ours with misaligned input, and The red box denotes the locally enlarged region for detail illustration).

As illustrated in Figure 15, although our model can preserve structural contours well, the misaligned inputs still cause slight edge blurring. Such spatial inconsistencies may undermine the reliability of feature aggregation by the FAM and introduce ghosting artifacts.

These limitations point out directions for future research. To mitigate sensor misalignment in engineering applications, future work could explore an end-to-end “registration-fusion” joint optimization paradigm to actively compensate for baseline parallax via cross-modal implicit addressing. Second, to overcome the limited effective receptive field of underlying networks, integrating state-space models like Mamba holds the promise of enhancing structural coherence in complex macro-scale scenes with lower computational complexity. Finally, exploring adaptive multi-scale frequency transformations will help broaden the framework’s generalization boundaries under unknown extreme meteorological conditions.

## 6. Conclusions

This paper proposes a scene-adaptive image fusion network (HL-Fuse) based on dynamic low-rank modulation and frequency-domain collaboration. Building upon a dual-branch feature decoupling framework, this method introduces a Hyper-LoRA dynamic low-rank modulation mechanism driven by a SceneHyperNet, enabling the base feature fusion process to adaptively adjust according to the input scene. Meanwhile, it integrates the FAM into the fused feature reconstruction stage, enhancing edge and texture representation by reweighting high- and low-frequency components. HL-Fuse can preserve infrared thermally salient targets while retaining the structural and textural information in visible images relatively well.

Experimental results on four public datasets, namely MSRS, TNO, M^3^FD, and FMB, demonstrate that HL-Fuse achieves superior or competitive performance on most objective metrics. Combined with a subjective user study, the model is verified to have favorable visual stability and subjective acceptability in complex scenarios, and can alleviate issues including brightness attenuation, edge blurring, and insufficient target saliency existing in some fusion methods to a certain extent. Special analysis on the scene-adaptive property further confirms that the modulation parameters generated by Hyper-LoRA change regularly with scene conditions, with genuine scene responsiveness, which supports the stable adaptation performance of the model across different environments. Ablation experiments and repeated validation with multiple random seeds show that Hyper-LoRA and FAM play complementary roles in the complete framework, and the conclusions are stable and reproducible: dynamic low-rank modulation improves the model’s adaptability to different scene inputs, the frequency-domain modulation module strengthens the high-frequency structural representation in fused features, and there exist reasonable performance trade-offs between modules on some metrics. Sensitivity analysis of key hyperparameters and efficiency benchmark tests also verify the rationality of the current configuration selection, and the model can achieve real-time inference while ensuring fusion performance. Downstream YOLOv5s object detection experiments further indicate that the fused images of HL-Fuse can provide effective inputs for high-level visual tasks, achieving sound performance on the person class and the overall comprehensive metric; the detection gains vary across different classes, reflecting that different objects have different degrees of dependence on thermal saliency, structural texture, and local boundary information in the fused images.

Overall, the experimental results indicate that HL-Fuse offers a promising solution for the fusion of infrared and visible images in complex all-weather perception and remote sensing applications.

## Figures and Tables

**Table 1:** Subjective visual preference of three fusion methods on different datasets.

Methods	MSRS	TNO	M^3^FD	FMB
SwinFusion	2.81	2.12	2.72	2.63
CDDFuse	2.36	2.64	2.73	2.55
Conti-Fuse	1.64	1.97	1.67	1.74
Ours	2.82	2.91	2.52	2.73

**Table 2:** Quantitative comparison results for the MSRS datasets (The best values are highlighted in bold, and the second-best values are underlined).

Methods	MSRS							
	EN	SD	AG	SF	MI	SCD	VIF	Qabf
DeFusion	6.46	37.91	2.77	8.60	2.32	1.33	0.76	0.53
LRRNet	6.19	31.76	2.64	8.47	2.03	0.79	0.54	0.45
SwinFusion	6.62	43.00	3.55	11.09	3.14	**1.69**	0.99	0.65
DATFuse	6.48	36.48	3.56	10.93	2.70	1.41	0.91	0.64
YDTR	5.65	25.37	2.19	7.40	1.92	1.14	0.58	0.35
CDDFuse	6.7 0	43.38	3.73	**11.56**	3.47	1.62	**1.05**	*0.69*
DDBFusion	5.97	28.42	2.78	8.55	1.53	1.34	0.63	0.38
Conti-Fuse	6.65	42.72	3.69	11.52	**3.69**	1.64	1.04	**0.71**
Ours	**6.74**	**43.75**	**3.76**	**11.56**	3.43	1.66	**1.05**	0.69

**Table 3:** Quantitative comparison results between the TNO datasets (the best values are highlighted in bold, and the second-best values are underlined).

Methods	TNO							
	EN	SD	AG	SF	MI	SCD	VIF	Qabf
DeFusion	6.65	33.19	2.77	6.79	1.86	1.51	0.56	0.38
LRRNet	6.90	38.57	3.63	9.44	1.76	1.53	0.54	0.37
SwinFusion	6.90	41.17	4.37	11.37	2.31	1.71	*0.76*	0.54
DATFuse	6.58	29.65	3.74	10.09	2.36	1.45	0.70	0.51
YDTR	6.42	28.25	2.85	8.01	1.69	1.50	0.60	0.40
CDDFuse	7.12	**46** **.00**	4.9 0	13.15	2.19	1.76	0.77	0.54
DDBFusion	6.62	36.13	4.31	11.71	1.42	1.72	0.61	0.41
Conti-Fuse	6.92	40.07	4.30	11.74	**3.1** **0**	1.57	**0.86**	**0.58**
Ours	**7.16**	44.48	**5.2** **0**	**13.82**	1.88	**1.8** **0**	0.71	0.52

**Table 4:** Quantitative comparison results between the M^3^FD datasets (the best values are highlighted in bold, and the second-best values are underlined).

Methods	M^3^FD							
	EN	SD	AG	SF	MI	SCD	VIF	Qabf
DeFusion	6.48	27.77	2.83	8.15	2.19	1.28	0.56	0.40
LRRNet	6.44	27.18	3.59	10.68	1.94	1.46	0.57	0.50
SwinFusion	6.79	35.84	4.60	13.69	2.88	1.56	0.77	0.61
DATFuse	6.40	26.31	3.43	10.47	2.86	1.29	0.64	0.49
YDTR	6.55	28.00	3.30	10.10	2.20	1.51	0.63	0.48
CDDFuse	6.9 0	37.24	4.86	14.78	2.71	1.65	0.79	0.61
DDBFusion	6.61	30.98	3.60	10.02	1.82	1.66	0.60	0.43
Conti-Fuse	6.77	34.55	4.51	14.19	**3.46**	1.38	0.79	0.61
Ours	**6.98**	**39.23**	**5.2** **0**	**15.7** **0**	2.59	**1.7** **0**	**0.81**	**0.62**

**Table 5:** Quantitative comparison results between the FMB datasets (the best values are highlighted in bold, and the second-best values are underlined).

Methods	FMB							
	EN	SD	AG	SF	MI	SCD	VIF	Qabf
DeFusion	6.31	27.11	2.47	8.00	2.24	1.24	0.60	0.46
LRRNet	6.28	26.26	3.08	10.18	2.08	1.27	0.60	0.55
SwinFusion	6.67	35.38	4.04	13.51	3.08	1.56	0.85	0.67
DATFuse	6.32	26.31	3.26	10.85	2.93	1.23	0.71	0.57
YDTR	6.47	28.53	2.89	9.73	2.35	1.42	0.67	0.51
CDDFuse	6.77	37.06	4.3 0	14.58	*3* *.00*	1.62	*0.88*	**0.68**
DDBFusion	6.56	31.73	3.00	9.40	2.05	1.55	0.61	0.44
Conti-Fuse	6.62	33.81	4.04	14.03	**3.55**	1.41	0.87	0.67
Ours	**6.86**	**38.31**	**4.57**	**15.36**	2.85	**1.67**	**0.9** **0**	**0.68**

**Table 6:** Quantitative comparison results of ablation experiments on the MSRS dataset (the best values are highlighted in bold, and the second-best values are underlined, and √ indicates that the component is used, while the blank indicates that it is not used).

Model	Baseline	FAM	Fixed-LoRA	Hyper-LoRA	EN	SD	AG	SF	MI	SCD	VIF	Qabf
$M_{1}$	√				6.65	43.16	3.70	11.5 0	3.32	1.64	1.02	0.68
$M_{2}$	√	√			6.66	43.24	3.69	11.45	3.18	1.67	1.02	0.68
$M_{3}$	√		√		6.70	43.21	3.70	11.46	3.25	1.68	1.03	**0.69**
$M_{4}$	√			√	6.71	43.01	3.66	11.32	3.32	**1.7** **0**	1.04	**0.69**
$M_{5}$	√	√	√		6.72	42.92	3.71	11.44	3.22	1.68	1.04	0.68
Ours	√	√		√	**6.74**	**43.75**	**3.76**	**11.56**	**3.43**	1.66	**1.05**	**0.69**

**Table 7:** Quantitative comparison of computational complexity and inference efficiency across different dynamic low-rank dimensions.

Rank	Params (M)	FLOPs (G)	VRAM (MB)	Inference Time (ms)	FPS
4	1.27	29.21	109.12	24.24	41.26
8	1.33	29.22	114.24	22.88	43.71
16	1.47	29.22	119.94	22.57	44.31
32	1.73	29.22	121.69	22.96	43.56

**Table 8:** Quantitative comparison results between the MSRS-Daytime datasets (the best values are highlighted in bold, and the second-best values are underlined).

Methods	MSRS-Daytime							
	EN	SD	AG	SF	MI	SCD	VIF	Qabf
DeFusion	7.15	47.28	3.69	10.71	2.62	1.16	0.71	0.57
LRRNet	6.93	40.72	3.62	10.75	2.46	0.94	0.61	0.54
SwinFusion	7.28	52.96	4.7	13.94	3.90	1.61	0.96	0.70
DATFuse	7.09	44.45	4.58	13.55	3.18	1.27	0.82	0.66
YDTR	6.56	34.78	3.21	10.05	2.34	1.29	0.65	0.48
CDDFuse	7.34	54.01	4.85	14.44	4.16	1.51	**0.99**	0.72
DDBFusion	6.56	35.74	3.69	10.87	1.65	1.39	0.55	0.38
Conti-Fuse	7.28	52.84	4.82	14.35	**4.31**	**1.55**	0.97	**0.73**
Ours	**7.38**	**54.48**	**4.9** **0**	**14.53**	4.18	1.53	0.98	0.72

**Table 9:** Quantitative comparison results between the MSRS-Nighttime datasets (the best values are highlighted in bold, and the second-best values are underlined).

Methods	MSRS-Nighttime							
	EN	SD	AG	SF	MI	SCD	VIF	Qabf
DeFusion	5.78	28.69	1.87	6.54	2.02	1.5	0.82	0.49
LRRNet	5.47	22.94	1.68	6.23	1.60	0.64	0.47	0.37
SwinFusion	5.98	*33.2* *0*	2.43	8.29	2.40	1.77	1.02	0.61
DATFuse	5.88	28.64	2.56	8.35	2.22	1.55	0.99	0.62
YDTR	4.74	16.12	1.18	4.80	1.50	0.99	0.51	0.22
CDDFuse	6.08	32.93	**2.64**	8.72	2.79	1.73	1.11	0.67
DDBFusion	5.39	21.22	1.88	6.27	1.42	1.29	0.70	0.39
Conti-Fuse	6.04	32.76	2.58	**8.73**	**3.09**	1.73	1.11	**0.69**
Ours	**6.12**	**33.21**	2.63	8.65	2.68	**1.78**	**1.12**	0.66

**Table 10:** Quantitative comparison results of object detection on the MSRS dataset (the best values are highlighted in bold, and the second-best values are underlined).

Methods	mAP@0.5			mAP@0.5:0.95		
	Person	Car	All	Person	Car	All
Infrared	0.822	0.829	0.826	0.449	0.506	0.478
Visible	0.633	0.847	0.740	0.290	0.556	0.423
DeFusion	0.837	0.837	0.837	0.483	0.547	0.515
LRRNet	0.827	0.826	0.827	0.468	0.549	0.509
SwinFusion	0.812	**0.85** **0**	0.831	0.449	**0.559**	0.504
DATFuse	0.833	0.827	0.830	0.476	0.545	0.511
YDTR	0.828	0.848	0.838	0.463	0.533	0.498
CDDFuse	0.836	0.838	0.837	0.482	0.536	0.509
DDBFusion	0.810	0.838	0.824	0.455	0.547	0.501
Conti-Fuse	0.819	0.841	0.830	0.455	0.553	0.504
Ours	**0.851**	0.846	**0.849**	**0.487**	0.558	**0.523**

## Data Availability

The raw questionnaire data supporting the findings of the subjective evaluation are available from the corresponding author upon reasonable request.

## Abbreviations

The following abbreviations are used in this manuscript:

HL-Fuse	Scene-Adaptive Image Fusion Network based on Hyper-LoRA and FAM
UAV	Unmanned aerial vehicle
CNNs	Convolutional neural networks
AE	Autoencoder
LoRA	Low-Rank Adaptation
Hyper-LoRA	Hypernetwork-driven Dynamic Low-Rank Modulation mechanism
FAM	Frequency-Aware Modulator
rFFT	Real Fast Fourier Transform
GAP	Global average pooling
MLP	Multilayer perceptron
MHSA	Multi-Head Self-Attention
SSIM	Structural Similarity Index Measure
EN	Entropy
SD	Standard deviation
AG	Average gradient
SF	Spatial frequency
MI	Mutual Information
SCD	Sum of correlations of differences
VIF	Visual information fidelity
Qabf	Visual Edge Information Transfer Factor
mAP	Mean average precision
VRAM	Video Random Access Memory
FPS	Frames per second
FLOPs	Floating-point operations
MSRS	Multi-Spectral Road Scenarios
M^3^FD	Multi-Modal Multi-Scene Fusion Dataset
TNO	Netherlands Organisation for Applied Scientific Research
FMB	Full-time Multi-modality Benchmark
YOLOv5s	You Only Look Once version 5 small

## Appendix A

This appendix contains supplementary data for our study on the proposed infrared and visible image fusion method. Specifically, Table A1 and Table A2 detail the training configurations of compared baseline methods and the quantitative results of the ablation study, respectively, while Figure A1 shows the visualized performance comparison of ablation models on representative evaluation metrics.

**Table A1:** Training Configurations and Input Specifications of All Compared Methods.

Methods	Training Dataset	Training Crop Size	Input Modality/Color Space	Weight Source
DeFusion	MS-COCO	256 × 256	IR (Gray) + VIS (RGB)	Official GitHub
LRRNet	MS-COCO	224 × 224	IR (Gray) + VIS (Gray)	Official GitHub
SwinFusion	TNO + RoadScene	120 × 120	IR (Gray) + VIS (Gray)	Official GitHub
DATFuse	TNO + RoadSence	120 × 120	IR (Gray) + VIS (Gray)	Official GitHub
YDTR	MSRS	128 × 128	IR (Gray) + VIS (RGB)	Official GitHub
CDDFuse	KAIST	128 × 128	IR (Gray) + VIS (Gray)	Official GitHub
DDBFusion	MS-COCO	224 × 224	IR (Gray) + VIS (Gray)	Official GitHub
Conti-Fuse	MSRS	256 × 256	IR (Gray) + VIS (RGB)	Official GitHub

**Table A2:** Quantitative Results of the Ablation Study (Mean ± Standard Deviation).

Model	EN	SD	AG	SF	MI	SCD	VIF	Qabf
$M_{1}$	6.71 ± 0.01	43.36 ± 0.30	3.71 ± 0.03	11.48 ± 0.08	3.25 ± 0.03	1.65 ± 0.04	1.04 ± 0.01	0.69 ± 0.01
$M_{2}$	6.69 ± 0.02	43.36 ± 0.33	3.71 ± 0.04	11.51 ± 0.08	3.28 ± 0.06	1.66 ± 0.04	1.03 ± 0.01	0.69 ± 0.01
$M_{3}$	6.70 ± 0.02	43.42 ± 0.27	3.72 ± 0.02	11.49 ± 0.04	3.25 ± 0.03	1.66 ± 0.02	1.04 ± 0.01	0.69 ± 0.01
$M_{4}$	6.70 ± 0.03	43.25 ± 0.27	3.72 ± 0.03	11.50 ± 0.05	3.33 ± 0.04	1.64 ± 0.02	1.04 ± 0.01	0.69 ± 0.00
$M_{5}$	6.70 ± 0.02	43.64 ± 0.54	3.74 ± 0.05	11.56 ± 0.13	3.19 ± 0.05	1.65 ± 0.06	1.03 ± 0.00	0.69 ± 0.01
Ours	6.68 ± 0.02	43.19 ± 0.13	3.71 ± 0.06	11.50 ± 0.12	3.30 ± 0.05	1.65 ± 0.03	1.03 ± 0.01	0.69 ± 0.00
